# TMEM59L promotes neuroendocrine differentiation and enzalutamide resistance in prostate cancer by regulating MUC1/β‐catenin/MYC axis

**DOI:** 10.1002/ctm2.70835

**Published:** 2026-09-16

**Authors:** Xiuchen Guo, Yifan Liu, Jiacheng Huang, Zhiwei Chen, Haihong Liao, Yulin Xu, Xingang Cui, Yongjiang Yu

**Affiliations:** ^1^ Department of Urology Xinhua Hospital, Shanghai Jiao Tong University School of Medicine Shanghai People's Republic of China; ^2^ Department of Urology School of Medicine Shanghai Children's Hospital Shanghai Jiao Tong University Shanghai People's Republic of China

**Keywords:** enzalutamide resistance, neuroendocrine differentiation, prostate cancer, scRNA‐seq, TMEM59L

## Abstract

**Background:**

Enzalutamide resistance in advanced prostate cancer often leads to treatment‐induced neuroendocrine prostate cancer (t‐NEPC), characterized by aggressive lineage plasticity. The molecular regulators governing neuroendocrine differentiation and phenotypic transition are not well understood. Here, we characterize the functional role of the transmembrane protein TMEM59L in driving PCa progression, lineage plasticity and therapeutic resistance.

**Methods:**

We combined bulk RNA sequencing, single‐cell RNA sequencing, and spatial transcriptomics datasets to assess TMEM59L expression patterns and their association with clinical features. We investigated the biological functions of TMEM59L using enzalutamide‐resistant (EnzR) PCa cell lines in vitro and corresponding mouse xenograft models in vivo. Mechanistic interactions were elucidated through molecular assays, including co‐immunoprecipitation (Co‐IP), GST pull‐down and molecular docking.

**Results:**

TMEM59L expression is negatively regulated by androgen receptor (AR) signalling and markedly elevated in EnzR PCa tissues. In PCa cells, ectopic TMEM59L expression induced a neuroendocrine‐like phenotype, enhanced stemness, promoted epithelial‐mesenchymal transition (EMT) and conferred enzalutamide resistance. Conversely, TMEM59L depletion restored drug sensitivity and significantly suppressed EnzR tumour growth in vivo. Mechanistically, TMEM59L directly interacts with MUC1 to facilitate its nuclear accumulation, thereby activating the downstream β‐catenin/MYC signalling axis. Importantly, pharmacological inhibition or genetic silencing of MUC1 effectively reversed TMEM59L‐induced neuroendocrine differentiation, stemness and enzalutamide resistance.

**Conclusions:**

Our findings uncover a previously unrecognized TMEM59L/MUC1/β‐catenin/MYC signalling cascade associated with neuroendocrine differentiation and enzalutamide resistance in PCa. Targeting this regulatory axis presents a promising therapeutic strategy to circumvent resistance to next‐generation AR signalling inhibitors.

## Highlights

1. Enzalutamide‐mediated AR suppression induces TMEM59L during prostate cancer progression.
2. TMEM59L interacts with MUC1 and promotes its nuclear enrichment.
3. Nuclear MUC1 activates β‐catenin/TCF4‐dependent MYC signalling, driving neuroendocrine differentiation, stemness and EMT.
4. TMEM59L depletion or MUC1‐C inhibition restores enzalutamide sensitivity and suppresses resistant tumour growth.

## Graphical abstract

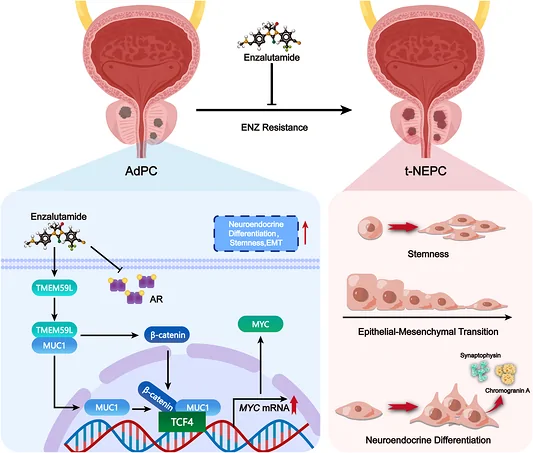

## 1 INTRODUCTION

Prostate cancer (PCa) continues to cause substantial morbidity and mortality worldwide, with treatment failure largely attributable to the emergence of resistant tumour states.^1^, ^2^ Androgen deprivation therapy (ADT) is a key aspect of clinical management for PCa due to its reliance on androgen receptor (AR) activity. Prolonged suppression of androgen signalling often results in castration‐resistant prostate cancer (CRPC), where tumour growth continues despite low androgen levels.^3^, ^4^, ^5^

Next‐generation AR signalling inhibitors (ARSIs) are extensively used to treat CRPC. However, their clinical benefit is constrained by acquired resistance, often associated with lineage plasticity and the development of treatment‐induced neuroendocrine prostate cancer (t‐NEPC).^6^, ^7^, ^8^ De novo neuroendocrine prostate cancer (NEPC) comprises less than 2% of new diagnoses, while treatment‐emergent NEPC (t‐NEPC) occurs in up to 17% of advanced metastatic castration‐resistant prostate cancer (mCRPC) cases following androgen receptor‐targeted therapy.^9^, ^10^ This aggressive phenotype is defined by a devastating 10% 5‐year survival rate.^8^, ^11^ At the molecular level, NEPC is commonly associated with diminished AR signalling, loss of the tumour suppressors p53 and RB, and activation of neural lineage regulators such as BRN2.^12^, ^13^ Enhanced stem‐like properties, epithelial–mesenchymal transition (EMT), and SOX2 induction also contribute to the neuroendocrine phenotype.^14^ Recent studies further highlight that NEPC pathogenesis and progression are driven by MYCN upregulation and increased expression of EZH2.^15^, ^16^, ^17^

Transmembrane proteins are increasingly recognized as key regulators of tumour progression and drug resistance. TMEM59L, a type‐I transmembrane protein, is primarily recognized for its functions in the central nervous system, particularly in regulating amyloid precursor protein (APP) trafficking and ATG16L1‐mediated non‐canonical autophagy.^18^, ^19^ Emerging evidence has begun to link TMEM59L to the progression of a limited number of malignancies, notably glioma and colorectal cancer.^20^, ^21^, ^22^ However, its role in AR signalling, neural lineage plasticity and therapy resistance in PCa remains largely unexplored.

In this study, we characterize TMEM59L as a pivotal nexus linking AR inhibition to a cascade of phenotypic reprogramming events, including neuroendocrine differentiation, EMT and stemness. Integrating multi‐omics datasets with functional assays, we demonstrate that TMEM59L expression is upregulated under therapeutic stress and serves as a critical driver of enzalutamide resistance and malignant progression. We establish that TMEM59L coordinates these aggressive phenotypes by fuelling a MUC1/β‐catenin/MYC signalling axis. By elucidating this targetable molecular cascade, our work uncovers a novel mechanism governing tumour cell plasticity, offering a strategic framework for therapeutic intervention in recalcitrant PCa.

## 2 MATERIALS AND METHODS

### 2.1 Cell lines and compounds

LNCaP cells (RRID: SCSP‐5021) were obtained from the Chinese Academy of Sciences Cell Bank. The DU145 (iCell‐h250), NCI‐H660 (iCell‐h515), 22Rv1 (iCell‐h230) and C4‐2 (iCell‐h626) cell lines were sourced from iCell Bioscience, China. Cell lines were authenticated using short tandem repeat analysis and regularly verified to be mycoplasma‐free. Enzalutamide‐resistant LNCaP cells (LNCaP EnzR) were generated according to a previously reported protocol.^23^ In brief, LNCaP cells were continuously cultured and passaged in medium containing enzalutamide for approximately 6 months. The drug concentration was gradually escalated from 1 to 10 µM during this period. Meanwhile, parental LNCaP cells were maintained in parallel cultures treated with DMSO as a vehicle control. LNCaP EnzR cells were passaged every 5–7 days and exposed to enzalutamide‐supplemented growth medium for 3–5 days in each passage. After 6 months of selection, the IC_50_ value of enzalutamide was determined to verify the successful development of the resistant cell line. Unless otherwise stated, cells were propagated in complete medium. The experiment shown in Figure 4I employed charcoal‐stripped fetal bovine serum (CSS) medium. Before exposure to dihydrotestosterone (DHT), LNCaP cells were adapted for 4 days to medium supplemented with CSS. Enzalutamide (S1250) was purchased from Selleck Chemicals (USA). DHT (GC19064) and GO‐203 (GC64914) were purchased from GlpBio (USA).

### 2.2 Clinical tissue specimens

Prostate adenocarcinoma tissues, preserved as formalin‐fixed, paraffin‐embedded samples, were retrospectively collected from Xinhua Hospital Affiliated to Shanghai Jiao Tong University School of Medicine. Eligibility required a documented pathological diagnosis, an available Gleason score, and sufficient preserved tumour tissue for histological assessment. Samples with inadequate or poorly preserved tumour regions were excluded. The final cohort comprised 86 cases, which were divided into three categories: GS < 7 (*n* = 13), GS = 7 (*n* = 28), and GS > 7 (*n* = 45).

### 2.3 Bioinformatics analysis

Human prostate single‐cell RNA‐sequencing (scRNA‐seq) count matrices and metadata were sourced from the NCBI Gene Expression Omnibus (GEO), specifically from datasets GSE137829,^24^ GSE141445,^25^ GSE150692,^26^ GSE172301,^27^ GSE172316,^27^ GSE176031,^28^ GSE181294,^29^ GSE185344^30^ and GSE278936.^31^ For GSE150692, only the human prostate samples were included. Disease status and tissue origin were assigned according to the metadata provided by the original studies and harmonized across datasets. Specifically, GSE137829 contributed six advanced tumours, including four CRPC and two mCRPC specimens; GSE141445 included 12 treatment‐naïve prostate cancer (TRNA) and one neoadjuvant androgen deprivation therapy (NEADT) sample; GSE150692 included two benign prostatic hyperplasia (BPH) and one adjacent non‐tumour sample; GSE172301 included 13 BPH samples; GSE172316 included six healthy prostate samples; GSE176031 included 27 TRNA and eight adjacent non‐tumour samples; GSE181294 included five healthy prostate, 18 TRNA, and 16 adjacent non‐tumour samples; GSE185344 included seven TRNA and seven adjacent non‐tumour samples; and GSE278936 included four BPH, 17 TRNA, 22 NEADT, five CRPC and four mCRPC samples. In total, 181 sample‐level profiles were included, comprising 11 healthy prostate, 19 BPH, 32 adjacent non‐tumour, 81 TRNA, 23 NEADT, nine CRPC and six mCRPC samples. Cell‐level quality control was performed separately for each dataset using Seurat. Cells were retained if they contained more than 500 but fewer than 6000 detected genes (nFeature_RNA > 500 and nFeature_RNA < 6000), fewer than 30 000 unique molecular identifier counts (nCount_RNA < 30 000), less than 20% mitochondrial transcripts, and less than 3% haemoglobin‐gene transcripts. Cells failing any of these criteria were excluded. The retained cells were subsequently subjected to normalization, data integration, dimensionality reduction, unsupervised clustering and cell‐type annotation based on established lineage‐specific markers.

Processed spatial transcriptomic expression matrices, spatial coordinates, and corresponding sample metadata were obtained from GSE278936. This dataset comprised 52 spatial transcriptomic profiles generated using the 10x Genomics Visium platform, including four BPH, 17 TRNA, 22 NEADT, five CRPC and four mCRPC samples. In the initial preprocessing workflow, genes present in less than five spatial spots and spots with fewer than 500 unique molecular identifier counts were removed. Subsequently, transcript counts were normalized using the scran method. The processed spatial transcriptomic data were used to evaluate TMEM59L expression across different disease and treatment states.

Processed and quality‐controlled bulk transcriptomic data were obtained from publicly available datasets. GSE78201 included 36 transcriptomic profiles from VCaP, LAPC‐4 and CWR‐R1 cells under control, 48‐h enzalutamide treatment, and enzalutamide‐resistant conditions.^32^ GSE88808 included 49 prostate tumours and 49 matched adjacent normal tissues.^33^ RNA‐sequencing data and clinical details for the TCGA Prostate Adenocarcinoma cohort (TCGA‐PRAD), comprising 499 primary tumours and 52 normal prostate tissues, were sourced from the Genomic Data Commons.^34^ The Beltran 2016 cohort included 49 advanced prostate cancer samples, comprising 34 CRPC adenocarcinomas and 15 neuroendocrine prostate cancer samples.^35^ In addition, GSE66187,^36^ the SU2C cohort^37^ and GSE126078^38^ contained transcriptomic profiles from 71 CRPC metastases obtained from 47 patients, 270 CRPC metastases, and 98 mCRPC tumours, respectively. Quality‐controlled and normalized expression matrices provided by the original studies were used for downstream analyses. Pathway‐level differences were assessed by gene set enrichment analysis using collections from MSigDB. A nominal *p* value below .05 was deemed to indicate significant enrichment. The direction and extent of pathway enrichment were represented by the normalized enrichment score.

### 2.4 Lentivirus production and plasmid transfection

The full‐length human *TMEM59L* CDS (NM_012109.3) was inserted into the pCDH lentiviral backbone by Servicebio for the generation of a stable overexpression cell line. Moreover, to achieve stable gene silencing, lentiviral knockdown vectors expressing shRNAs against *TMEM59L* or *MUC1*, along with a non‐targeting control shRNA, were synthesized by Tsingke Biotech; the specific sequences are detailed in Table S1. The shRNA constructs were packaged in HEK293T cells with the GMeasy system (Genechem). Viral supernatants harvested 48 and 72 h after transfection were clarified by filtration and subsequently concentrated by centrifugation. For transient interaction‐interface experiments, expression plasmids encoding FLAG‐tagged wild‐type TMEM59L (FLAG‐TMEM59L‐WT) and His‐tagged wild‐type MUC1 (His‐MUC1‐WT), together with the corresponding alanine‐substitution mutants FLAG‐TMEM59L‐C326A and His‐MUC1‐Y1218A, were generated by ObioTechnology. 293T cells were co‐transfected with specified combinations of wild‐type or mutant plasmids following the manufacturer's guidelines. FLAG‐empty vector (FLAG‐EV) and His‐empty vector (His‐EV) were included as negative controls. Forty‐eight hours after transfection, a portion of each cell lysate was retained as the input fraction, whereas the remainder underwent reciprocal precipitation with anti‐FLAG or anti‐His antibodies and subsequent immunoblot detection.

### 2.5 Tumour xenografts

To investigate TMEM59L's role in tumour resistance, 4‐week‐old male nude mice from GemPharmatech, China, received subcutaneous injections of 6.0 × 10^6^ LNCaP EnzR cells expressing either shTMEM59L or shNC expression. Cells were suspended in an equal volume of Matrigel, and five mice were included in each group. After tumour implantation, the animals were surgically castrated and orally treated with enzalutamide at 10 mg/kg/day. For rescue experiments, 22Rv1 cells stably expressing shNC, sh*TMEM59L* or sh*TMEM59L*+oe*TMEM59L* were implanted under identical conditions. All animal maintenance, monitoring and enzalutamide dosing protocols remained consistent across all experimental groups. Four‐week‐old male nude mice were subcutaneously implanted with LNCaP cells that stably expressed either TMEM59L or an empty vector to assess the pharmacological reversal of TMEM59L‐mediated resistance. Animals were randomly allocated to EV plus vehicle, EV plus enzalutamide, TMEM59L plus enzalutamide, or TMEM59L plus combined enzalutamide and GO‐203. Enzalutamide was administered orally at 10 mg/kg, whereas GO‐203 was delivered intraperitoneally at 18 mg/kg. All mice underwent surgical castration prior to the initiation of drug treatments. Tumour volumes and body weights were monitored regularly. To evaluate the TMEM59L/MUC1‐C axis in enzalutamide‐resistant tumours, LNCaP‐EnzR cells stably expressing shNC or shTMEM59L were implanted into mice. The mice were assigned to six groups: (1) shNC+DMSO, (2) shTMEM59L+DMSO, (3) shNC+GO‐203, (4) shNC+enzalutamide, (5) shTMEM59L+enzalutamide, and (6) shNC+GO‐203+enzalutamide. Enzalutamide was administered orally at 10 mg/kg once daily, and GO‐203 was administered intraperitoneally at 18 mg/kg according to the indicated treatment schedule. At the experimental endpoint, the tumours were excised, photographed, weighed, and processed for histopathological and immunohistochemical analyses.

### 2.6 Immunohistochemistry

Xenograft tumours were fixed in 10% neutral‐buffered formalin for 24 h, embedded in paraffin, and sectioned to a thickness of 4 µm. Human clinical specimens were prepared at the same section thickness. Histopathological features were evaluated using H&E staining on xenograft sections from all six treatment groups. For immunohistochemistry, the sections underwent deparaffinization, rehydration, antigen retrieval and blocking prior to being incubated with the primary antibodies. Human tissues were evaluated for TMEM59L, while xenografts were stained for TMEM59L, MUC1, CHGA, SYP, ENO2, β‐catenin and MYC. Immunoreactivity was detected with 3,3′‐diaminobenzidine and counterstained with haematoxylin. The median TMEM59L immunohistochemical score for the entire clinical cohort was used to classify specimens as TMEM59L‐high or TMEM59L‐low. The distribution of these categories across the three Gleason‐score groups was compared using Fisher's exact test. In xenograft sections, marker‐positive cells and cells showing nuclear MUC1 or β‐catenin staining were quantified in randomly chosen, non‐overlapping fields. Field‐level measurements were averaged to generate one representative value for each tumour.

### 2.7 RNA extraction, reverse transcription and quantitative real‐time PCR

PCa cells underwent total RNA extraction using TRIzol, following the manufacturer's guidelines. Two micrograms of RNA were reverse‐transcribed into cDNA with M‐MLV reverse transcriptase. Real‐time PCR was performed using a CFX Opus system. Transcript levels were normalized to GAPDH, with primer sequences detailed in Table S2.

### 2.8 Western blot analysis

Proteins were equally loaded, separated by SDS‐PAGE, and transferred to nitrocellulose membranes. To prevent nonspecific binding, samples were incubated for 1 h at room temperature in a solution of 5% skimmed milk in PBS with 0.1% Tween‐20. The membranes were incubated with the specified primary antibodies overnight at 4°C. The following antibodies were used: rabbit anti‐TMEM59L (PH3861; Abmart); rabbit anti‐β‐tubulin (2146; CST); rabbit anti‐CHGA (85798; CST); rabbit anti‐HDAC1 (ab7028; Abcam); rabbit anti‐EGFR (4267; CST); rabbit anti‐SYP (36406; CST), rabbit anti‐ENO2 (24330; CST), rabbit anti‐CD44 (37259; CST), rabbit anti‐CD133 (64326; CST), rabbit anti‐E‐Cadherin (3195; CST), rabbit anti‐N‐Cadherin (13116; CST), rabbit anti‐Lamin B1 (13435; CST), rabbit anti‐MUC1 (ab109185; Abcam), rabbit anti‐MYC (9402; CST), rabbit anti‐β‐Catenin (8480; CST), rabbit anti‐MMP‐7 (3801; CST), rabbit anti‐Cyclin D1 (2978; CST), rabbit anti‐GST (2625; CST), rabbit anti‐FLAG (14793; CST), rabbit anti‐HIS (12698; CST), rabbit anti‐SOX2 (2748; CST), rabbit anti‐ASCL1 (10585; CST) and rabbit anti‐BRN2 (12137; CST) for Western blotting. After secondary‐antibody incubation, immunoreactive bands were detected using chemiluminescence and captured with a ChemiDoc MP system.

### 2.9 Co‐immunoprecipitation (Co‐IP) assay

Each reaction involved incubating 1.5 mg of cellular protein overnight with 1 µg of the specified antibody and 100 µL of protein A/G agarose under rotation. Immune complexes were rinsed twice with 1 mL of a buffer comprising 10 mM HEPES (pH 7.9), 1 mM EDTA, 150 mM NaCl, and 1% Nonidet P‐40. The agarose‐bound material was eluted by heating in 60 µL of SDS sample buffer for 10 min, followed by separation on 6% polyacrylamide gels, transfer to nitrocellulose and analysis using the specified antibodies.

### 2.10 Glutathione S‐transferase (GST) pull‐down assay

Recombinant GST and GST‐tagged TMEM59L were equilibrated at room temperature for 20 minutes in a binding buffer containing 10 mM Tris‐HCl (pH 7.4), 50 mM NaCl, 5% glycerol, 0.1% Triton X‐100, and 0.1 mM DTT. This protein solution was then mixed with glutathione agarose 4B resin and rotated for 2 h. Bound proteins were recovered by elution with SDS loading buffer after three successive washes. SDS‐PAGE was used to separate the eluted fractions, which were further evaluated by immunoblotting.

### 2.11 EdU cell‐proliferation assay

DNA synthesis was evaluated with a Beyotime EdU assay kit. Cells were plated in 12‐well plates at a density of 1 × 10^4^ cells per well and incubated for 24 h, followed by a 30‐min exposure to 500 µL of 10 µM EdU. Cells were fixed with 4% paraformaldehyde for 15 minutes, permeabilized with 0.3% Triton X‐100 for 15 min, and then underwent click‐chemistry labelling for 30 min at room temperature in the dark. Nuclei were subsequently counterstained with Hoechst dye.

### 2.12 Immunofluorescence

Cells were cultured on poly‐D‐lysine‐coated coverslips, transfected for 72 h, and subjected to puromycin selection for another 72 h. Subsequently, they were fixed with 4% paraformaldehyde for 15 min, permeabilized using 0.1% Triton X‐100 for 15 min, and blocked with 5% FBS in PBS for 30 min. Following an overnight incubation with primary antibodies at 4°C, an Alexa Fluor 594‐conjugated secondary antibody was applied for 1 h in the dark. DAPI was used to label nuclei, and fluorescence images were acquired on a Leica microscope. Human prostate cancer tissue sections (4‐µm) underwent deparaffinization, rehydration, antigen retrieval and blocking for multiplex immunofluorescence staining. Separate sections were stained with antibody panels against AMACR/P504S, p63, and TMEM59L or against TMEM59L, MUC1, ENO2 and SYP, followed by detection using spectrally distinct fluorescence channels. Nuclei were counterstained with DAPI, and merged images were used to assess the spatial distributions of the specified proteins.

### 2.13 Migration and invasion assays

Transwell inserts were used to assess cell migration and invasion. For invasion experiments, the inserts were coated with Matrigel before the cells were added. Cells that migrated through the membrane were fixed with 4% paraformaldehyde and stained with crystal violet after 48–72 h of incubation.

### 2.14 Cell counting kit 8 (CCK 8) assay

PCa cells were seeded in 96‐well plates at a density of 5000 cells per well, with each experimental condition having three technical replicates. Following treatment, the medium was substituted with 100 µL of medium containing 10% CCK‐8 reagent. Plates were incubated at 37°C in the dark for 1 h, after which absorbance at 450 nm was measured using a microplate reader and normalized to vector control values.

### 2.15 Colony formation assay

Cells were seeded into 6‐well plates at a density of 500 cells per well and maintained in culture for 2 weeks. Fresh medium was supplied at regular intervals. At the endpoint, colonies were rinsed, fixed, stained with 0.1% crystal violet and counted.

### 2.16 Sphere formation assay

Sphere formation was assessed by seeding 500 cells per well in 6‐well ultra‐low‐attachment plates. Cells were cultured in serum‐free DMEM/F12 supplemented with 2% B27, 20 ng/mL EGF, 20 ng/mL bFGF, 0.4% BSA, and 5 µg/mL insulin. On Day 10, the spheres were photographed and quantified.

### 2.17 Statistical analysis

Quantitative results are presented as the mean ± standard deviation from three independent experiments, unless stated otherwise. Analyses were conducted in GraphPad Prism 9.0. Two‐tailed Student's *t*‐tests were used for two‐group comparisons, while one‐way ANOVA with suitable post hoc tests was applied for datasets with three or more groups. Survival differences were evaluated using Kaplan–Meier analysis. A two‐sided *p*‐value of 0.05 or less was considered statistically significant.

## 3 RESULTS

### 3.1 TMEM59L is upregulated in EnzR prostate cancer and responds to androgens

**FIGURE 1:**
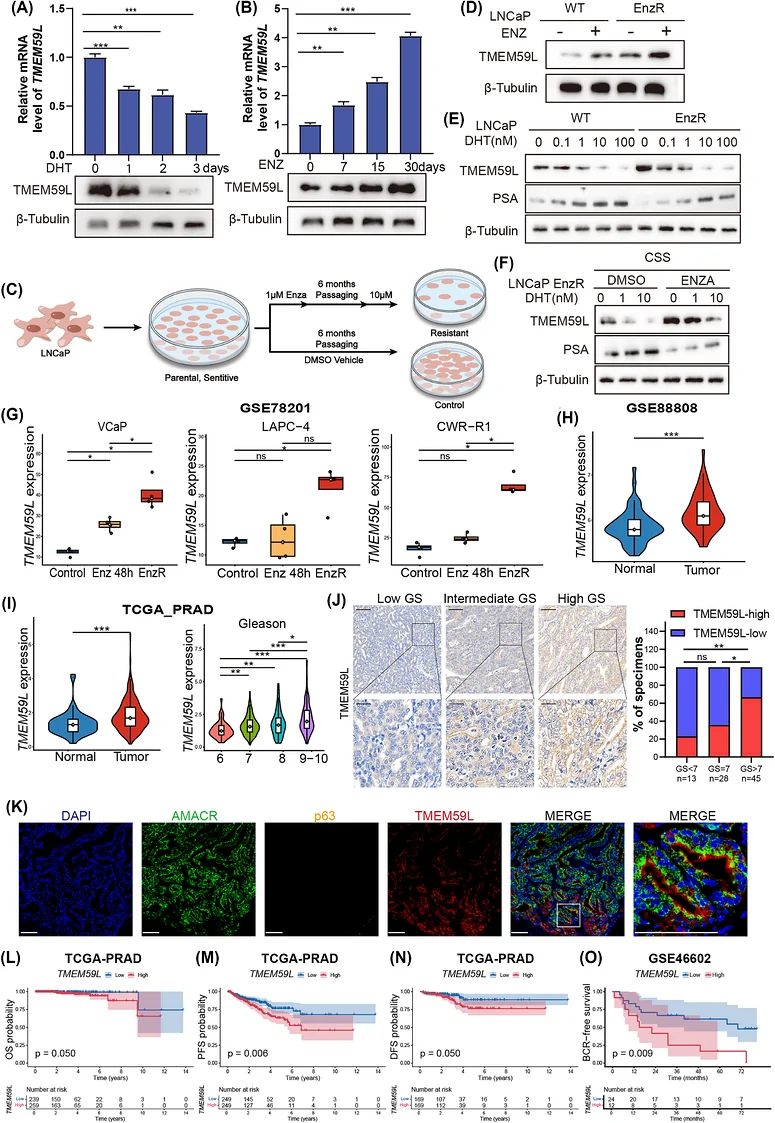
The expression of TMEM59L is upregulated in enzalutamide‐resistant PCa. (A) LNCaP cells were cultured in CSS medium for 4 days before challenged with 1 nM DHT for 0–3 days. The mRNA and protein expression levels of TMEM59L were measured by qRT‐PCR and Western blotting assays. CSS, charcoal‐stripped serum; DHT, dihydrotestosterone. (B) LNCaP cells were treated with 10 µM enzalutamide for 0–30 days. TMEM59L mRNA and protein levels were detected at the indicated time points by qRT‐PCR and Western blotting. ENZ, enzalutamide. (C) Schematic diagram representing the establishment of LNCaP EnzR cell lines. LNCaP parental cells were treated with enzalutamide (1 µM to 10 µM) for 6 months. LNCaP parental cells were cultured in parallel. (D) Western blot was used to test the protein levels of TMEM59L in both WT and EnzR LNCaP cells, with or without Enz treatment. Cells were exposed to 10 µM Enz or a DMSO vehicle control for 48 h. (E) Parental and EnzR LNCaP cells were cultured in CSS medium and treated with the indicated concentrations of DHT (0, 0.1, 1, 10 and 100 nM) for 3 days. TMEM59L and PSA protein levels were assessed by Western blotting. (F) LNCaP EnzR cells cultured in CSS medium were treated with DHT (0, 1, or 10 nM) in the presence or absence of 10 µM Enz for 3 days. TMEM59L and PSA protein levels were examined by Western blotting. β‐Tubulin served as the loading control for all Western blot analyses. (G and H) The relative gene expression of *TMEM59L* is compared between control and EnzR groups in the GSE78201 (G) dataset and between normal prostate tissues and PCa samples in the GSE88808 (H) dataset. (I) Violin plots showing *TMEM59L* mRNA expression in the TCGA‐PRAD cohort. Left, comparison between normal prostate tissues and primary prostate tumours. Right, comparison among tumours stratified by Gleason score (6, 7, 8 and 9–10). (J) Representative immunohistochemical staining of TMEM59L in human prostate cancer specimens stratified by Gleason score (GS): low GS (GS < 7, *n* = 13), intermediate GS (GS = 7, *n* = 28), and high GS (GS > 7, *n* = 45). Boxed regions in the upper panels are shown at higher magnification in the corresponding lower panels. Scale bars, 100 µm (upper panels) and 25 µm (lower panels). The stacked bar graph shows the proportions of TMEM59L‐high and TMEM59L‐low specimens in each group. (K) Representative multiplex immunofluorescence staining of human prostate cancer tissue for DAPI (blue), AMACR/P504S (green), p63 (orange), and TMEM59L (red). Merged images show TMEM59L expression predominantly within AMACR‐positive/p63‐negative malignant epithelial regions. The boxed region is shown at higher magnification in the rightmost panel. Scale bars, 100 µm. (L–O) Kaplan–Meier curves to show the correlation between *TMEM59L* expression levels and patients’ prognosis from the TCGA_PRAD and GSE46602 datasets.

The androgen‐AR signalling pathway is pivotal to the progression of PCa. This study initially investigated the responsiveness of TMEM59L to androgen stimulation. In LNCaP cells, Dihydrotestosterone (DHT) treatment led to a time‐dependent decrease in TMEM59L expression at both mRNA and protein levels (Figure 1A); conversely, enzalutamide treatment significantly upregulated its expression (Figure 1B). To examine TMEM59L expression in enzalutamide‐resistant (EnzR) PCa, an EnzR cell line was established via six‐month exposure of LNCaP cells to escalating enzalutamide doses (Figure 1C), followed by phenotypic validation (Figure S1A–C). Molecular analysis revealed that LNCaP EnzR cells maintained full‐length AR expression and had elevated AR‐V7 levels compared to parental cells, indicating that AR‐V7 upregulation might play a role in resistance in this model (Figure S1D).

TMEM59L expression was notably higher in LNCaP EnzR cells than in parental cells, with enzalutamide treatment further amplifying this increase (Figure 1D). Subsequent experiments demonstrated that DHT reduced TMEM59L protein expression in LNCaP EnzR cells in a dose‐dependent manner (Figure 1E), whereas enzalutamide counteracted this suppression when used in combination (Figure 1F). Consistent with these findings, Gene Set Enrichment Analysis (GSEA) of the TCGA_PRAD database showed a significant negative correlation between androgen response signalling activity and *TMEM59L* expression (Figure S1E). The data suggest that AR signalling negatively regulates TMEM59L, which is significantly upregulated in enzalutamide resistance.

Bioinformatic analysis of multiple datasets (GSE78201, GSE168668 and GSE33316) confirmed that *TMEM59L* expression is significantly elevated in EnzR cell lines and androgen‐deprived xenograft tumours (Figure 1G and Figure S1F–H). Additionally, *TMEM59L* expression was higher in PCa tissues than in benign tissues across the GSE88808 and TCGA_PRAD cohorts (Figure 1H,I), a finding corroborated by western blotting of 12 pairs of clinical specimens (Figure S1I). Within the TCGA‐PRAD cohort, TMEM59L expression increased with Gleason score and was also associated with higher PSA levels, advanced T stage, and lymph‐node involvement (Figure 1I and Figure S1J). To further validate these observations at the tissue level, we performed TMEM59L immunohistochemistry in an independent cohort of 86 prostate cancer specimens. The percentage of specimens with high TMEM59L expression rose progressively from low to intermediate to high Gleason score groups, with a notably higher proportion in the high‐score group compared to the other groups (Figure 1J). Multiplex immunofluorescence further showed that TMEM59L was predominantly localized within AMACR‐positive/p63‐negative malignant epithelial regions, confirming its expression in prostate adenocarcinoma cells (Figure 1K). Elevated TMEM59L mRNA levels correlated with notably reduced overall survival (OS), disease‐free survival (DFS), progression‐free survival (PFS), and biochemical recurrence‐free (BCR‐free) survival in both the TCGA_PRAD (Figure 1L–N) and GSE46602 (Figure 1O) cohorts. TMEM59L is inversely regulated by AR signalling, elevated in EnzR PCa, and associated with advanced clinicopathological characteristics and unfavourable clinical outcomes.

### 3.2 TMEM59L contributes to EnzR and promote EnzR tumour progression

**FIGURE 2:**
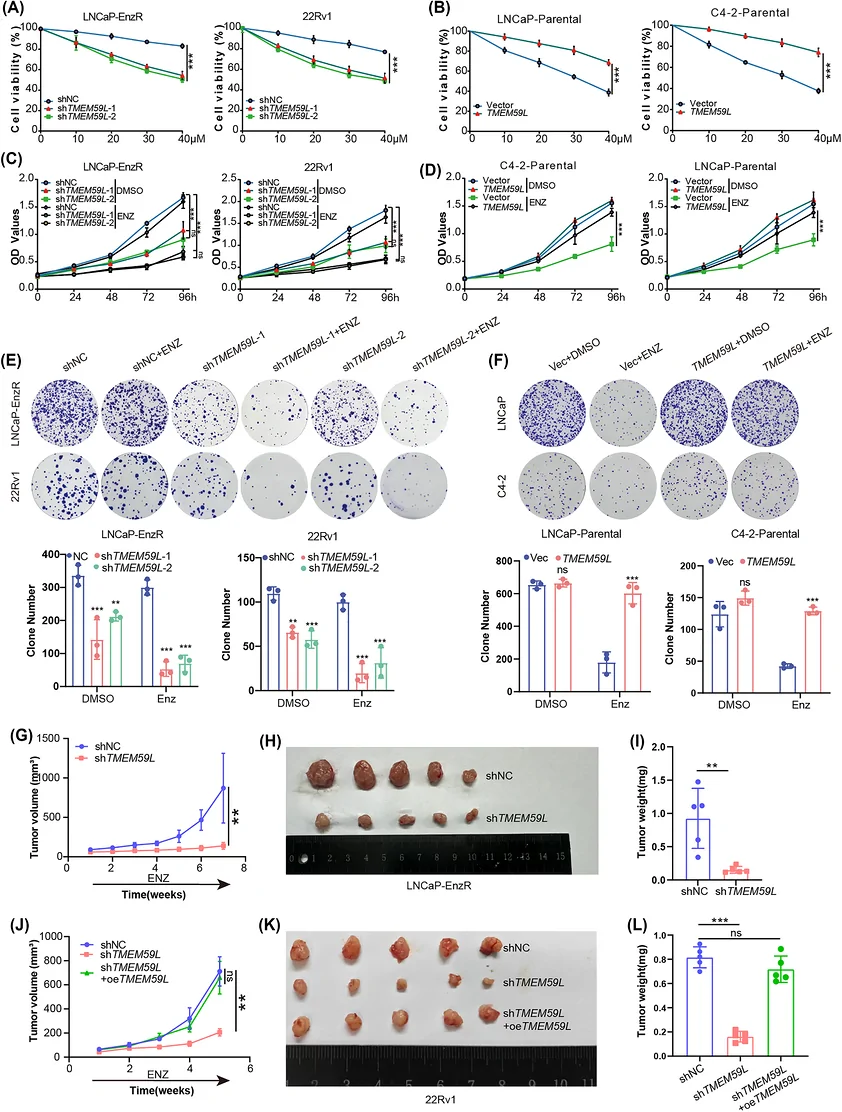
TMEM59L contributes to EnzR and promotes EnzR tumour progression. (A and B) Cell viability measured by CCK‐8 assay in the indicated cell lines under enzalutamide treatment. LNCaP‐EnzR and 22Rv1 cells were transfected with shNC or TMEM59L shRNA (shTMEM59L, stable transfection) (A) and LNCaP‐Parental and C4‐2‐Parental cells were transfected with empty vector (Vec) or TMEM59L overexpression plasmid (TMEM59L) (B). (C–F) Cell proliferation assessed by CCK‐8 assay and colony formation assay in indicated cells. For colony formation assays, colonies containing > 50 cells were counted and plotted. Quantitative analysis of colony numbers was shown in the panel below (E and F). ENZ, 20 µM enzalutamide. (G–I) Xenograft PCa tumour growth upon TMEM59L depletion. LNCaP‐EnzR cells with stable knockdown of control or sh*TMEM59L* were injected subcutaneously into nude mice (*n*  =  5). Tumour size was measured once every week after nude mice were castrated (G). At the endpoint, tumours isolated from euthanized mice were weighed and photographed (H, I). (J–L) Xenograft PCa tumour growth upon TMEM59L depletion and rescue. 22Rv1 cells with stable expression of shNC, sh*TMEM59L*, or sh*TMEM59L*+oe*TMEM59L* were injected subcutaneously into nude mice (*n* = 5). Tumour size was measured once every week after nude mice were castrated (J). At the endpoint, tumours isolated from euthanized mice were photographed (K) and weighed (L). Data are represented as mean ± SD of three independent experiments. *p < .05, **p < .01, ***p < .001.

To investigate the functional importance of *TMEM59L* in prostate tissues, we analysed CRISPR‐Cas9 knockout screening data from the DepMap portal. TMEM59L gene effect scores were uniformly negative in both prostate cancer and non‐malignant or transformed prostate cell lines (Figure S2A). These widespread negative scores indicate that *TMEM59L* depletion universally inhibits cell growth or induces cell death in these models. To determine whether TMEM59L contributes to the development of EnzR in PCa, we established stable *TMEM59L*‐knockdown EnzR cell models and *TMEM59L*‐overexpressing parental models (Figure S2B,C). Cytotoxicity assays demonstrated that *TMEM59L* depletion significantly resensitized resistant cells to enzalutamide, whereas its overexpression conferred resistance to the drug in parental cells (Figure 2A,B). CCK‐8 and colony formation assays demonstrated that TMEM59L knockdown, alongside enzalutamide treatment, decreased the proliferation rate and clonogenic potential of EnzR cells (Figure 2C,E). In contrast, *TMEM59L* overexpression protected parental cells from enzalutamide‐induced growth suppression (Figure 2D,F). These findings were further corroborated by EdU incorporation assays (Figure S2D). To validate the role of TMEM59L in tumour growth in vivo, male BALB/c nude mice were subcutaneously inoculated with 6.0  ×  10^6^ LNCaP EnzR cells expressing either sh*TMEM59L* or shNC, followed by surgical castration and daily oral administration of enzalutamide (10 mg/kg). *TMEM59L* knockdown led to significantly lower tumour volumes and weights compared to the control group (Figure 2G–I). To further confirm these findings and verify target specificity, we performed a rescue experiment using 22Rv1 xenografts under identical enzalutamide treatment conditions. Consistent with the LNCaP‐EnzR model, *TMEM59L* depletion markedly suppressed 22Rv1 tumour growth. Crucially, the exogenous re‐expression of TMEM59L completely reversed this tumour‐suppressive phenotype, restoring both tumour volumes and final weights to levels comparable to the control group (Figure 2J–L). Taken together, our data demonstrate that *TMEM59L* facilitates EnzR progression by conferring a robust survival advantage to PCa cells during anti‐androgen therapy.

### 3.3 TMEM59L‐related transcriptional patterns identification by scRNA‐seq data

**FIGURE 3:**
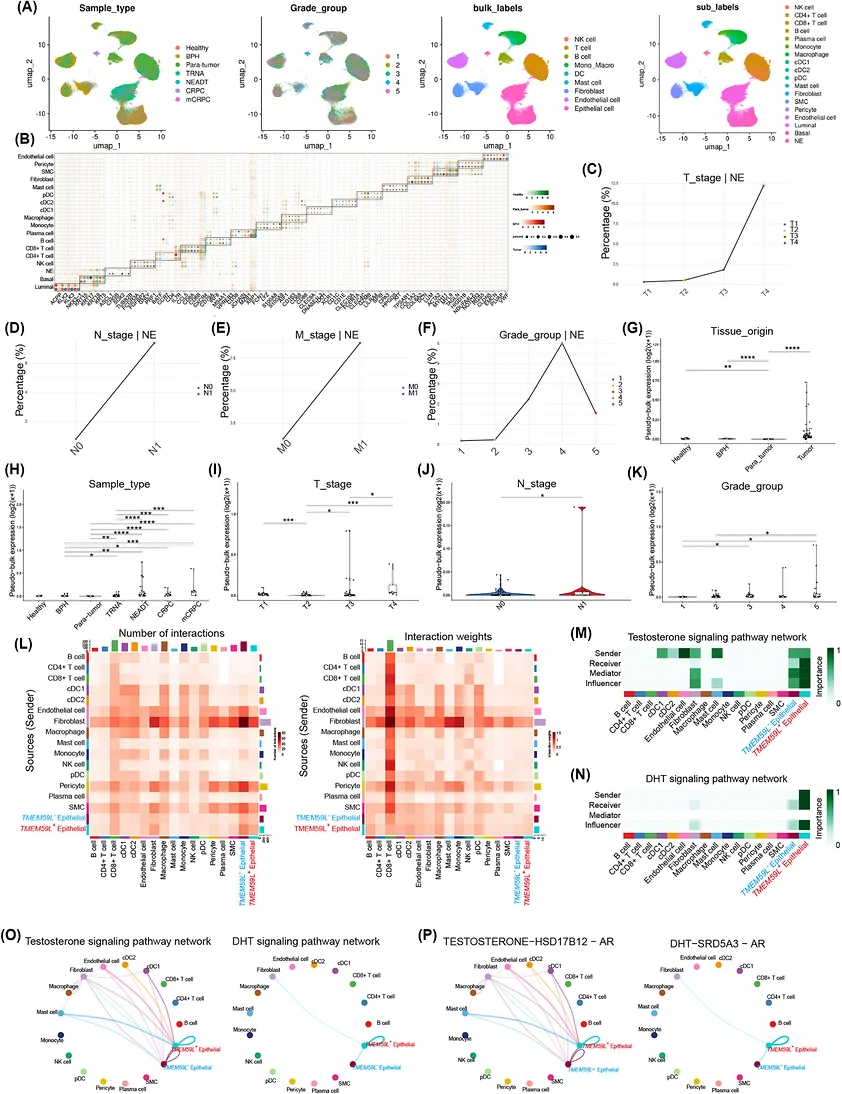
TMEM59L‐related transcriptional patterns identification by scRNA‐seq data. (A) UMAP visualization of integrated single‐cell RNA‐seq data. Cells are coloured by sample type, Gleason grade group, bulk cell‐type annotations, and refined subcluster annotations, respectively. (B) Dot plot showing scaled expression of representative marker genes across annotated cell types. Dot size represents the proportion of cells expressing each gene, and colour intensity indicates average expression level. (C–F) Percentage of NE in primary tumours with different T stages (C), N stages (D), M stages (E) and Grade groups (F). (G–K) Pseudo‐bulk TMEM59L expression (log2(x+1)) comparisons across clinical and pathological categories. Stratification by tissue origin (Healthy, BPH, Para‐tumour, Tumour) (G), disease states (Healthy, BPH, Para‐tumour, TRNA, NEADT, CRPC, mCRPC) (H); T stages (T1‐4) (I), N stages (N0‐1) (J) and Gleason grade groups (1–5) (K). Statistical significance is indicated as *p < .05, **p < .01, ***p < .001, ****p < .0001. (L) Heatmaps summarizing the number of interactions (left) and interaction weights (right) between sender and receiver cell types. Rows represent sender cells and columns represent receiver cells. Colour intensity indicates interaction frequency or strength. (M and N) Signalling role analysis for the Testosterone (M) and DHT (N) signalling pathways. Heatmaps display the relative importance of each cell type as Sender, Receiver, Mediator, or Influencer within the inferred signalling networks. (O) Visualization of cell‐type‐specific communication networks for Testosterone (left) and DHT (right) signalling pathways. Directed edges indicate inferred ligand–receptor interactions among cell populations. (P) Ligand–receptor pair‐specific interaction networks. Left: Testosterone–HSD17B12–AR axis. Right: DHT–SRD5A3–AR axis. Arrows indicate directionality from ligand‐producing cells to AR‐expressing target cells, highlighting androgen receptor–mediated intercellular communication.

After confirming that TMEM59L exhibits oncogenic properties associated with Enz resistance and PCa progression, we further investigated the downstream molecular mechanisms underlying its resistance‐promoting effects. To this end, we integrated single‐cell sequencing data from nine datasets (GSE137829, GSE141445, GSE150692, GSE172301, GSE172316, GSE176031, GSE181294, GSE185344 and GSE278936), comprising 181 patient samples: 11 healthy controls, 19 benign prostatic hyperplasia samples (BPH), 32 para‐tumour samples, 81 treatment‐naïve prostate cancer samples (TRNA), 23 neoadjuvant endocrine therapy–treated samples (NEADT), 9 CRPC samples, and 6 mCRPC samples. After quality control and preprocessing, a total of 386 664 cells were retained for downstream analysis. Cells were classified based on sample type, pathological grade (Grades 1–5), T stage (T1–T4), N stage (N0–1), and M stage (M0–1). Using classical marker genes, the cells were categorized into major populations: NK cells, CD4^+^ T cells, CD8^+^ T cells, B cells, plasma cells, monocytes, macrophages, cDC1, cDC2, pDC, mast cells, fibroblasts, smooth muscle cells (SMC), pericytes, endothelial cells, luminal cells, basal cells, and neuroendocrine (NE) cells. These populations were visualized using UMAP dimensionality reduction (Figure 3A and Figure S3A). Dot‐plot analysis further validated the accuracy of the cell‐type annotations by demonstrating cell‐type–specific marker gene expression patterns (Figure 3B and Figure S3B). Although these cell types were present across different disease stages, their relative proportions varied substantially. Stacked bar‐plot analysis revealed dynamic shifts in cellular composition during tumour progression (Figure S3C). NE cells were increasingly prevalent in tumours with advanced T stage, lymph node involvement (N1), distant metastasis (M1), and higher Grade groups (Figure 3C–F), suggesting a strong link between NE differentiation and aggressive disease.

We next examined the expression pattern of the hub gene *TMEM59L* across cell populations and found that it was predominantly enriched in epithelial cells, particularly within NE and luminal subsets (Figure S3D,E). Pseudo‐bulk analysis indicated that TMEM59L expression was markedly elevated in tumour tissues compared to non‐malignant tissues (Figure 3G). TMEM59L expression was higher in NEADT and mCRPC than in TRNA but did not differ significantly among NEADT, CRPC and mCRPC (Figure 3H). Its expression showed statistically significant differences in selected comparisons across T stages and Grade groups and was significantly higher in N1 than in N0 disease, although no consistent stepwise increase was observed across T stages or Grade groups 2–5 (Figure 3I–K). These cross‐sectional findings associate TMEM59L with tumour development, treatment adaptation and selected aggressive features.

Given the high expression of *TMEM59L* in epithelial cells, epithelial populations were stratified into *TMEM59L* positive (*TMEM59L* ^+^) and *TMEM59L* negative (*TMEM59L* ^−^) subgroups. CellChat analysis revealed extensive communication between these epithelial subsets and other components of the tumour microenvironment (Figure 3L and Figure S3F). Focusing on androgen signalling, network centrality analysis showed that *TMEM59L* ^+^ epithelial cells occupied a more central role in testosterone and DHT signalling networks (Figure 3 M,N). Pathway‐specific analysis indicated that endothelial cells, fibroblasts, and mast cells transmitted testosterone‐related signals towards *TMEM59L* ^+^ epithelial cells, whereas DHT signalling involved fewer interactions, mainly with *TMEM59L* ^+^ epithelial cells acting as signal senders (Figure 3O and Figure S3G). Ligand–receptor analysis further identified prominent communication through the TESTOSTERONE–HSD17B12–AR and DHT–SRD5A3–AR axes (Figure 3P). To determine whether these communication features were retained in advanced disease, cells from the mCRPC samples were separately extracted and subjected to CellChat analysis. The mCRPC‐specific analysis revealed extensive interactions between TMEM59L^+^ and TMEM59L^−^ epithelial cells and multiple stromal and immune cell populations (Figure S3H). Notably, TMEM59L^+^ epithelial cells exhibited the highest relative importance as senders, receivers, mediators and influencers within the testosterone signalling network (Figure S3I). These findings indicate that TMEM59L is linked to specific androgen‐related intercellular communication patterns in epithelial cells, including within the mCRPC microenvironment.

### 3.4 TMEM59L promotes neuroendocrine differentiation in prostate cancer

**FIGURE 4:**
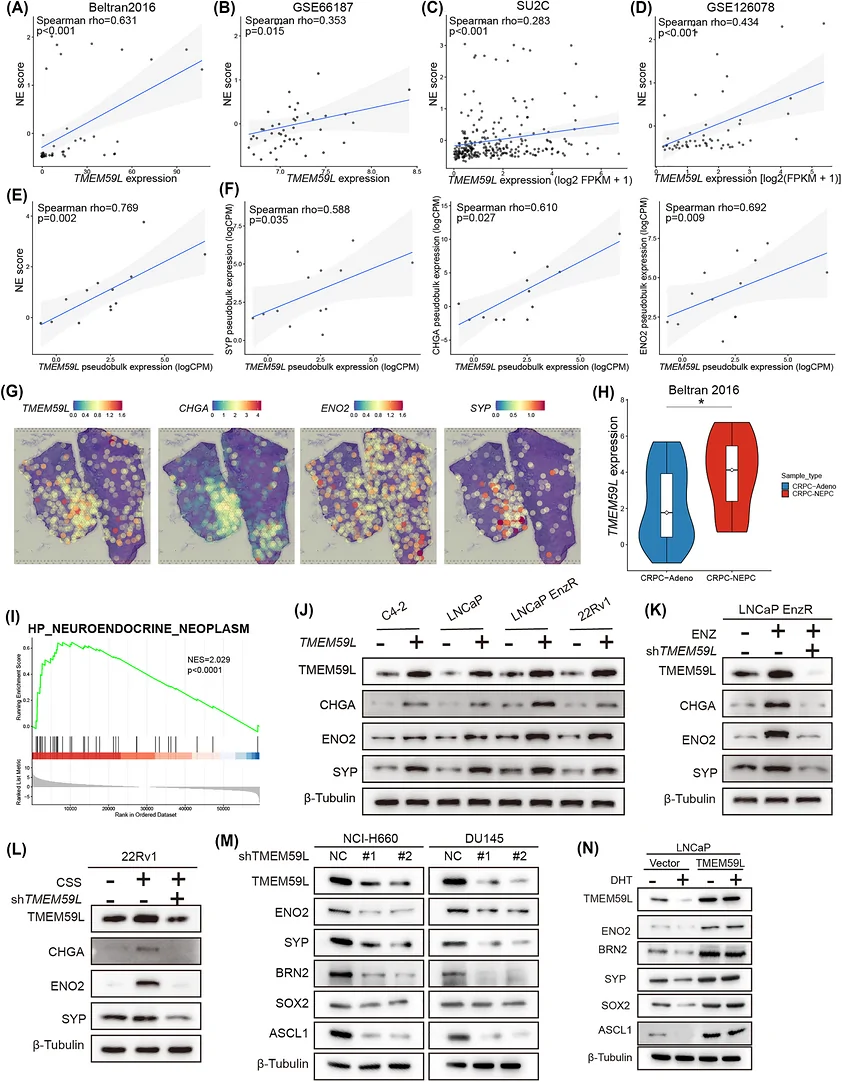
TMEM59L promotes neuroendocrine differentiation in prostate cancer. (A–D) Spearman correlation analyses between TMEM59L expression and the neuroendocrine (NE) score in the Beltran 2016 (A), GSE66187 (B), SU2C (C), and GSE126078 (D) cohorts. The NE scores were calculated using the mRNA Z score of ten NE signature genes (SYP, CHGA, NKX2‐1, PCSK1, SCN3A, CHGB, SCG3, CHRNB2, ENO2 and ELAVL4). Blue lines represent linear regression fits. (E) Correlation between pseudobulk TMEM59L expression and the NE score. (F) Correlations of pseudobulk TMEM59L expression with the neuroendocrine marker genes SYP, CHGA and ENO2. (G) Spatial transcriptomic analysis showing co‐localization of TMEM59L with neuroendocrine markers (CHGA, SYP, ENO2). Feature intensity is represented by colour gradients. (H) TMEM59L expression in the Beltran 2016 cohort comparing CRPC‐Adeno and CRPC‐NEPC samples. Violin plots show significantly higher TMEM59L expression in CRPC‐NEPC tumours (*p* < .05). (I) GSEA of the HP_NEUROENDOCRINE_NEOPLASM signature in tumours, demonstrating significant enrichment of neuroendocrine‐related gene programs in tumours with high TMEM59L expression. (J) Western blot analysis of TMEM59L and neuroendocrine markers (CHGA, ENO2, SYP) in prostate cancer cell lines (C4‐2, LNCaP, LNCaP EnzR and 22Rv1) with TMEM59L overexpression. β‐Tubulin serves as loading control. (K) Western blot analysis in LNCaP EnzR cells under ENZ treatment with or without shTMEM59L. TMEM59L knockdown attenuates ENZ‐induced neuroendocrine marker expression. (L) Western blot analysis in 22Rv1 cells under CSS conditions with or without shTMEM59L. TMEM59L knockdown reduces androgen deprivation‐induced neuroendocrine marker expression. (M) Western blot analysis of TMEM59L, the neuroendocrine markers ENO2 and SYP, and the lineage‐plasticity regulators BRN2, SOX2, and ASCL1 in NCI‐H660 and DU145 cells transduced with a non‐targeting control shRNA (NC) or two independent TMEM59L‐targeting shRNAs (#1 and #2). β‐Tubulin served as the loading control. (N) Western blot analysis of TMEM59L, the neuroendocrine markers ENO2 and SYP, and the lineage‐plasticity regulators BRN2, SOX2 and ASCL1 in vector‐control and TMEM59L‐overexpressing LNCaP cells treated with or without DHT. β‐Tubulin served as the loading control.

We initially examined several independent prostate cancer cohorts to explore the link between TMEM59L and neuroendocrine differentiation. TMEM59L expression showed a positive correlation with the NE score across multiple cohorts: Beltran 2016 (Spearman ρ = 0.631, *p* < .001), GSE66187 (ρ = 0.353, *p* = .015), SU2C (ρ = 0.283, *p* < .001), and GSE126078 (ρ = 0.434, p < .001) (Figure 4A–D). Tumour epithelial‐cell expression profiles from single‐cell RNA‐sequencing datasets of CRPC and mCRPC samples were consistently aggregated at the sample level for pseudobulk analysis. The analysis identified a strong positive correlation between TMEM59L expression and the NE score (ρ = 0.769, *p *= .002), along with significant positive correlations with SYP (ρ = 0.588, *p* = .035), CHGA (ρ = 0.610, *p* = .027), and ENO2 (ρ = 0.692, *p* = .009) expression (Figure 4E,F). In addition, at the individual‐cell level, TMEM59L expression showed a modest but significant negative correlation with AR activity. The Beltran 2016 and TCGA‐PRAD cohorts further validated the positive correlations between TMEM59L and specific NE markers (Figure S4B). TMEM59L expression varied across spatial transcriptomic samples (Figure S4C), and spatial mapping demonstrated that TMEM59L‐enriched regions overlapped with areas enriched for CHGA, ENO2 and SYP expression (Figure 4G). In the Beltran 2016 cohort, TMEM59L expression was notably elevated in CRPC‐NEPC compared to CRPC‐Adeno tumours (Figure 4H). Moreover, GSEA revealed significant enrichment of the HP_NEUROENDOCRINE_NEOPLASM signature in tumours with high TMEM59L expression (Figure 4I). These findings consistently associate elevated TMEM59L expression with neuroendocrine‐related transcriptional programmes across bulk, single‐cell and spatial transcriptomic datasets.

We next investigated whether TMEM59L functionally regulates neuroendocrine‐associated features. TMEM59L overexpression increased CHGA, ENO2, and SYP expression in C4‐2, LNCaP, LNCaP EnzR and 22Rv1 cells (Figure 4J). In LNCaP EnzR cells, ENZ treatment elevated TMEM59L and NE marker expression, while TMEM59L knockdown significantly reduced the ENZ‐induced increase in CHGA, ENO2 and SYP expression (Figure 4K). Similarly, under androgen‐depleted CSS conditions, TMEM59L silencing in 22Rv1 cells suppressed the induction of CHGA and ENO2 and reduced SYP expression (Figure 4L). In the AR‐independent NCI‐H660 and DU145 models, two independent TMEM59L‐targeting shRNAs broadly reduced the NE markers ENO2 and SYP, together with the lineage‐plasticity regulators BRN2, SOX2 and ASCL1 (Figure 4 M). Conversely, TMEM59L overexpression in LNCaP cells increased these NE‐associated markers and lineage regulators, and this effect remained evident in the presence of DHT (Figure 4N). Together, these results support a functional role for TMEM59L in promoting and maintaining neuroendocrine differentiation in prostate cancer.

### 3.5 TMEM59L drives the EMT and cancer stemness of PCa

**FIGURE 5:**
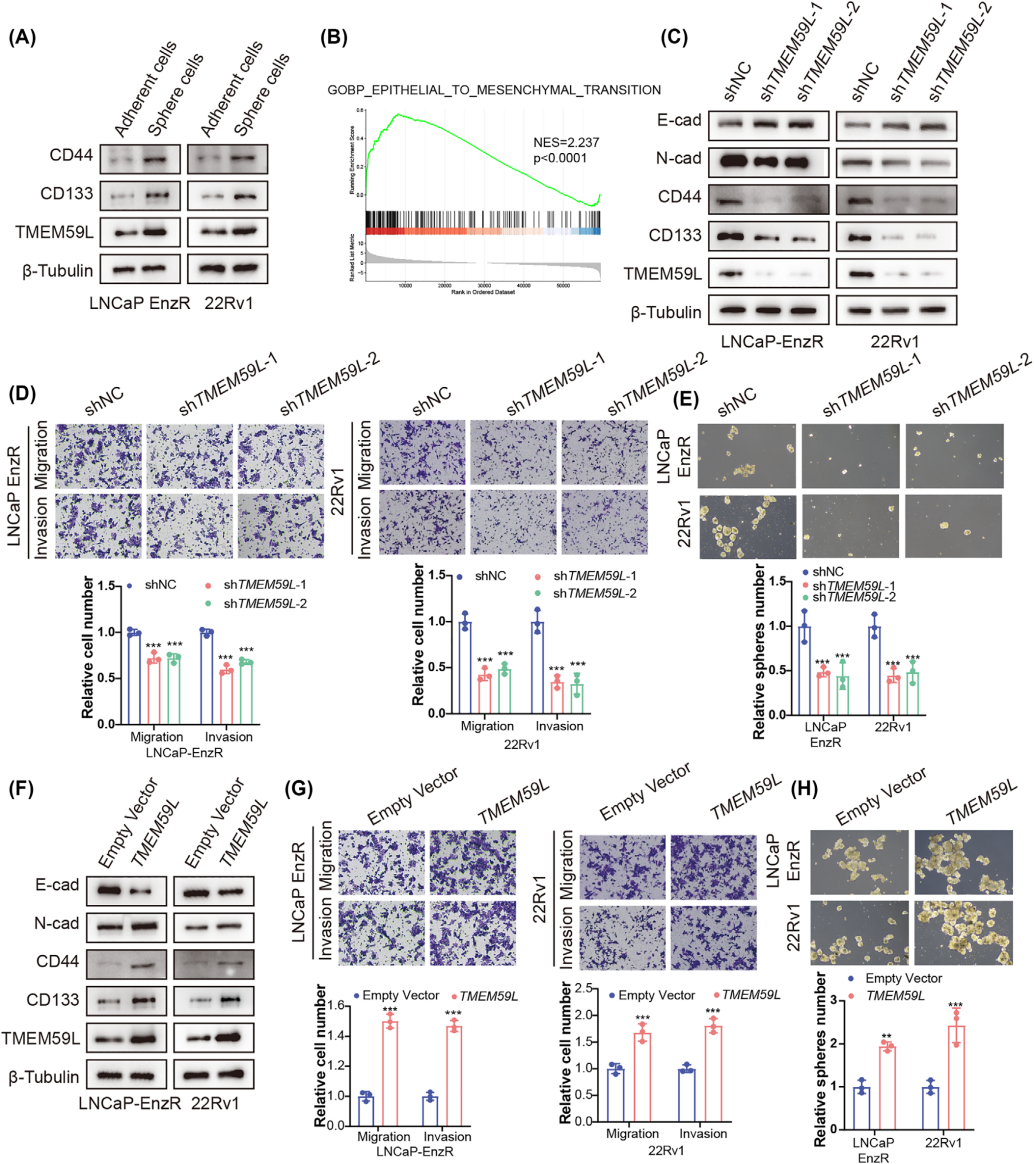
TMEM59L drives the EMT and cancer stemness of PCa. (A) The expressions of TMEM59L, CD44 and CD133 in tumourspheres and adherent Pca cells (LNCaP EnzR and 22Rv1) were compared by Western blotting. (B) GSEA of genes ranked by TMEM59L expression demonstrating enrichment of the EMT hallmark gene set. (C) Western blotting presented the expressions of CD44, CD133, E‐cadherin and N‐cadherin in PCa cells transfected with shNC or shTMEM59L. (D) Transwell assays were performed to examine the potential migration and invasion of TMEM59L knockdown cells or negative control cells. Representative images (upper panels) and quantification of relative cell numbers (lower panels) are shown. (E) The ability of sphere formation was examined by tumoursphere assay in TMEM59L knockdown cells or negative control cells. Representative images (upper panels) and quantification of relative sphere numbers (lower panels) are shown. (F) Western blotting presented the expressions of CD44, CD133, E‐cadherin and N‐cadherin in PCa cells transfected with empty vector or TMEM59L overexpression plasmid. (G) Transwell assays were performed to examine the potential migration and invasion of PCa cells transfected with empty vector or TMEM59L overexpression plasmid. Representative images (upper panels) and quantification of relative cell numbers (lower panels) are shown. (H) Tumoursphere assay was employed to determine the ability of sphere formation in PCa cells transfected with empty vector or TMEM59L overexpression plasmid. Representative images (upper panels) and quantification of relative sphere numbers (lower panels) are shown. Quantitative data are presented as mean ± SD from at least three independent experiments. Comparisons between two groups were performed using unpaired two‐tailed Student's *t*‐test. For multiple‐group comparison, one‐way ANOVA followed by post hoc multiple comparison testing was applied. *p < .05, **p < .01, ***p < .001.

EMT and stemness‐related pathways contribute to tumour lineage plasticity in PCa, which in turn facilitates NE differentiation and the emergence of NEPC.^14^ To determine if TMEM59L‐mediated NE differentiation is coupled with PCa cell stemness, we performed sphere formation assays. We found that TMEM59L and stemness markers were significantly enriched in LNCaP EnzR and 22Rv1 spheres compared to adherent cells (Figure 5A). Furthermore, GSEA of the TCGA‐PRAD cohort confirmed a robust positive correlation between *TMEM59L* levels and EMT signalling signatures (Figure 5B). To validate these associations, we silenced *TMEM59L* in PCa cells. This led to a marked reduction in stemness markers (CD133, CD44) and the mesenchymal marker N‐cadherin, alongside an upregulation of E‐cadherin (Figure 5C). Functional assays further confirmed that *TMEM59L* depletion significantly attenuated the migratory, invasive, and sphere‐forming capacities of PCa cells (Figure 5D,E). Conversely, ectopic expression of TMEM59L increased the levels of CD133, CD44, and N‐cadherin, while concurrently suppressing E‐cadherin (Figure 5F). Correspondingly, *TMEM59L* overexpression markedly enhanced the migration, invasion and sphere‐forming potential of both cell lines (Figure 5G,H). Collectively, these findings position TMEM59L as a central orchestrator of EMT and stemness programs, which are essential for the acquisition of aggressive NE features.

### 3.6 TMEM59L interacts with MUC1 and promotes its nuclear accumulation

**FIGURE 6:**
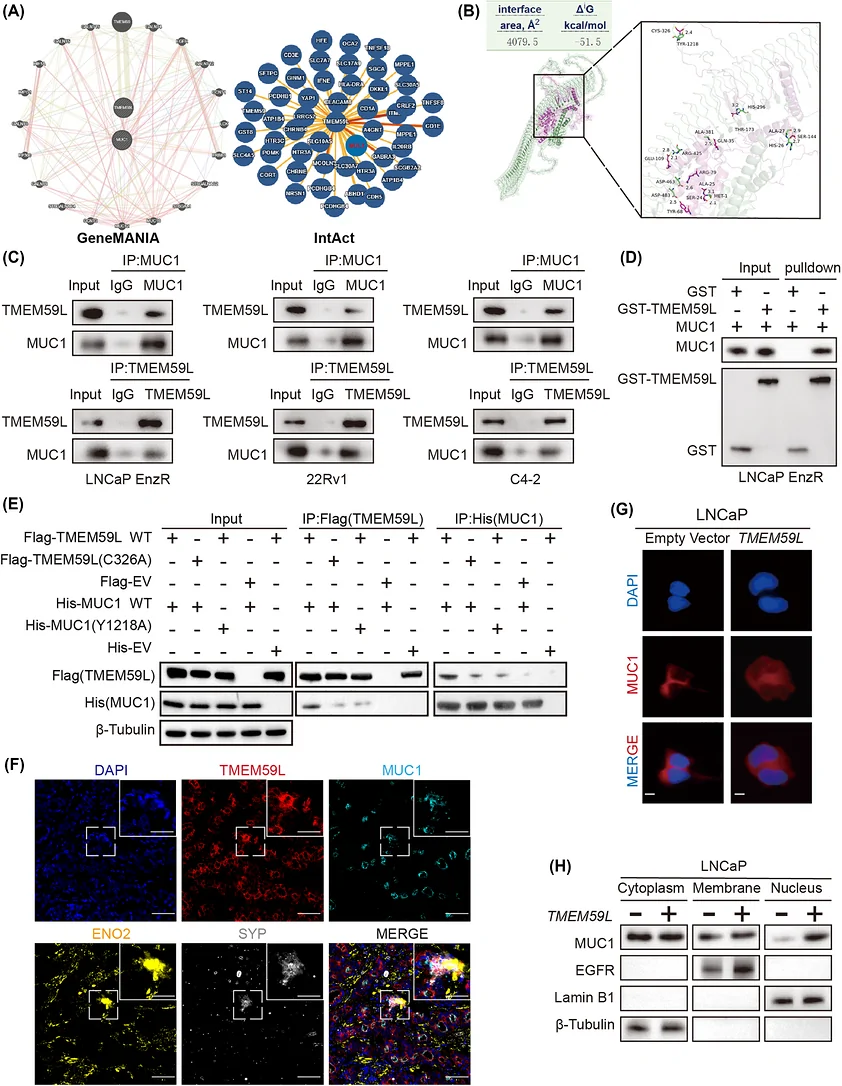
TMEM59L interacts with MUC1 and promotes its nuclear accumulation. (A) Interaction Network of TMEM59L constructed by GeneMANIA and IntAct. (B) Visualization of protein‐protein docking results of TMEM59L with MUC1. (C) Co‐immunoprecipitation (Co‐IP) assays demonstrating endogenous interaction between MUC1 and TMEM59L in LNCaP EnzR, 22Rv1 and C4‐2 cells. Cell lysates were immunoprecipitated with anti‐MUC1 or anti‐TMEM59L antibodies, followed by immunoblotting with the indicated antibodies. IgG was used as a negative control. (D) GST pulldown assay to detect the interaction between TMEM59L and MUC1. MUC1 was pulled down by GST‐TMEM59L immobilized on GST agarose beads but not by GST. (E) Reciprocal co‐immunoprecipitation assays evaluating the interaction between FLAG‐tagged TMEM59L (WT or C326A mutant) and His‐tagged MUC1 (WT or Y1218A mutant). Cells were co‐transfected with the indicated constructs, with FLAG‐empty vector (FLAG‐EV) and His‐empty vector (His‐EV) serving as negative controls. Cell lysates were immunoprecipitated with anti‐FLAG or anti‐His antibodies and immunoblotted with the indicated antibodies. (F) Representative multiplex immunofluorescence staining of human prostate cancer tissue for DAPI (blue), TMEM59L (red), MUC1 (cyan), ENO2 (yellow), and SYP (white). Boxed regions are shown at higher magnification in the insets. Scale bars, 50 µm in the main images and 25 µm in the insets. (G) LNCaP cells were transfected with TMEM59L overexpression plasmid for 72 h, followed by puromycin selection for another 72 h, and then subjected to immunofluorescence staining using MUC1 antibodies. Scale bar, 10 µm. (H) LNCaP cells were transfected with TMEM59L overexpression plasmid for 72 h and selected for 72 h before being subjected to subcellular fractionation and WB analysis. Tubulin, EGFR, and Lamin B1 were used as cytoplasmic, membrane and nuclear protein loading controls, respectively.

To investigate how TMEM59L promotes enzalutamide resistance and NE differentiation in PCa, we queried the intAct and GeneMANIA databases, identifying Mucin 1 (MUC1) as a potential interactor (Figure 6A). Since MUC1 is a known positive regulator of enzalutamide resistance and NE differentiation,^17^, ^39^ we hypothesized that TMEM59L promotes PCa progression via a MUC1‐dependent pathway. Molecular docking using GRAMM predicted a stable protein complex between TMEM59L and MUC1 (Figure 6B), an association subsequently validated by co‐immunoprecipitation (Co‐IP) assays in cellulo (Figure 6C). In vitro GST pull‐down assays further confirmed the direct nature of this binding (Figure 6D). To further characterize the interaction interface, molecular docking identified a close contact between TMEM59L Cys326 and MUC1 Tyr1218, with a predicted inter‐residue distance of approximately 2.4 Å (Figure 6B). We therefore generated FLAG‐tagged TMEM59L C326A and His‐tagged MUC1 Y1218A mutants and evaluated their binding capacities using reciprocal co‐immunoprecipitation assays. Substitution of either TMEM59L Cys326 or MUC1 Tyr1218 with alanine substantially weakened the interaction compared with the corresponding wild‐type proteins (Figure 6E). Because residual binding remained detectable, these residues appear to contribute importantly to, rather than solely determine, formation of the TMEM59L–MUC1 complex. We next examined the spatial relationship among TMEM59L, MUC1, and neuroendocrine markers in human prostate cancer tissue. Multiplex immunofluorescence revealed spatial overlap of TMEM59L with MUC1, ENO2, and SYP within the same tumour regions, providing tissue‐level support for the association of TMEM59L with neuroendocrine‐associated features (Figure 6F).

To explore how TMEM59L regulates MUC1, immunofluorescence assays were conducted in LNCaP cells overexpressing *TMEM59L* and the vector control group. We found that *TMEM59L* overexpression significantly increased nuclear MUC1 levels (Figure 6G), a finding corroborated by subcellular fractionation and Western blotting using EGFR, Lamin B1, and Tubulin as membrane, nuclear and cytoplasmic markers, respectively (Figure 6H). Collectively, these findings identify MUC1 as a direct TMEM59L‐binding partner and indicate that TMEM59L promotes the nuclear accumulation of MUC1. These findings establish a molecular foundation for MUC1's role in TMEM59L‐linked enzalutamide resistance and neuroendocrine phenotypes.

### 3.7 TMEM59L alters EnzR and NE differentiation via MUC1/β‐catenin/MYC signalling

Having established the interaction between TMEM59L and MUC1, we next investigated whether MUC1 is functionally required for TMEM59L‐mediated enzalutamide resistance and neuroendocrine differentiation. Because crosstalk between MUC1 and AR signalling has previously been reported, we first examined the relationship between MUC1 expression and AR pathway activity. DHT treatment decreased MUC1 expression while increasing the canonical AR target PSA, whereas enzalutamide increased MUC1 expression and concomitantly reduced PSA (Figure S4A,B). Furthermore, shMUC1 effectively reduced MUC1 expression but increased both total AR and PSA protein levels (Figure S4C). These reciprocal expression patterns suggest a negative regulatory relationship between MUC1 and AR signalling.

**FIGURE 7:**
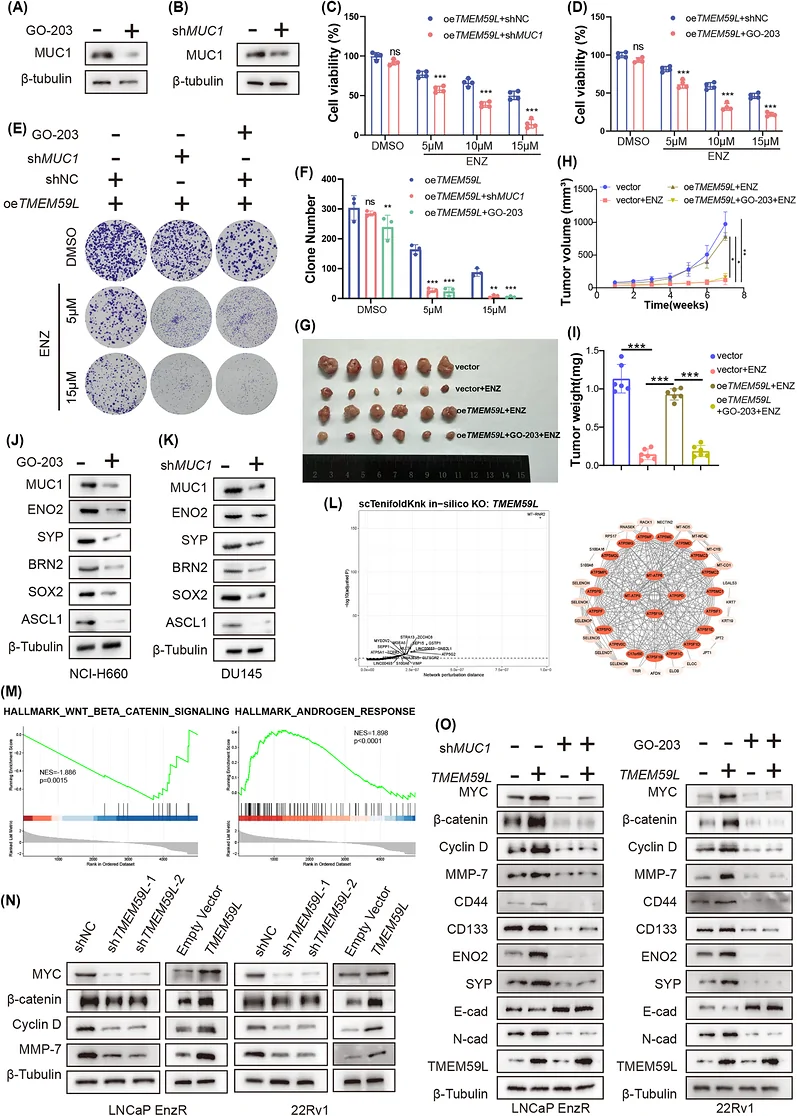
TMEM59L alters EnzR and NE differentiation via MUC1/β‐catenin/MYC signalling. (A) MUC1 protein expression in LNCaP cells treated with 10 µM GO‐203. (B) MUC1 protein expression in LNCaP cells transfected with shNC or MUC1 shRNA. (C) LNCaP cells cotransfected with the indicated shRNA and overexpression plasmids for 48 h were treated with DMSO or enzalutamide (5, 10 or 15 µM) for 24 h. Cell viability was assayed by CCK‐8 assays. (D) LNCaP cells transfected with overexpression plasmids for 48 h were treated with 10 µM GO‐203 for 48 h and then treated with DMSO or enzalutamide (5, 10 or 15 µM) for 24 h. Cell viability was assayed by CCK8 assays. (E and F) Colony formation assays of LNCaP cells with TMEM59L overexpression in combination with shMUC1 or GO‐203 treatment under DMSO or ENZ (5 µM and 15 µM) conditions. Representative images (E) and quantification of colony numbers (F) are shown. (G) Representative images of xenograft tumours derived from indicated treatment groups. (H) Tumour growth curves showing tumour volume over time in nude mice (*n*  =  6) bearing control or TMEM59L‐overexpressing tumours treated with vehicle, ENZ, or ENZ plus GO‐203. (I) Tumour weights at endpoint. (J) Western blot analysis of MUC1, the neuroendocrine markers ENO2 and SYP, and the lineage‐plasticity regulators BRN2, SOX2, and ASCL1 in NCI‐H660 cells treated with or without the MUC1‐C inhibitor GO‐203. (K) Western blot analysis of MUC1, ENO2, SYP, BRN2, SOX2 and ASCL1 in DU145 cells transduced with control or shMUC1. β‐Tubulin served as the loading control. (L) scTenifoldKnk in silico knockout analysis of TMEM59L based on single‐cell RNA‐seq data. Left: transcriptional perturbation landscape upon simulated TMEM59L deletion. Right: reconstructed gene regulatory network highlighting TMEM59L‐associated regulatory modules. (M) GSEA performed using the TMEM59L‐regulated gene network identified in (L), demonstrating significant enrichment of Wnt/β‐catenin signalling and androgen response pathways. (N) Western blotting presented the expressions of MYC, β‐catenin, Cyclin D and MMP7 in PCa cells transfected with shTMEM59L or TMEM59L overexpression plasmid. (O) Western blot analysis of MYC/β‐catenin signalling components, stemness markers (CD44, CD133), neuroendocrine markers (ENO2, SYP), and EMT markers (E‐cadherin and N‐cadherin) in LNCaP EnzR and 22Rv1 cells with TMEM59L overexpression combined with shMUC1 or GO‐203 treatment. β‐Tubulin served as a loading control.

We assessed MUC1's functional role by genetically silencing it with shMUC1 or inhibiting it pharmacologically using the MUC1‐C inhibitor GO‐203, confirming the effectiveness of both methods via Western blot analysis (Figure 7A,B). In TMEM59L‐overexpressing LNCaP cells, MUC1 knockdown or GO‐203 treatment significantly enhanced the inhibitory effects of increasing concentrations of enzalutamide on cell viability (Figure 7C,D). Consistently, both interventions markedly reduced the clonogenic survival of TMEM59L‐overexpressing cells under enzalutamide treatment (Figure 7E,F). In LNCaP EnzR cells, enzalutamide alone exerted only limited effects on colony formation and proliferation, whereas its combination with GO‐203 substantially suppressed both phenotypes (Figure S4D–F). These findings indicate that MUC1 inhibition restores enzalutamide responsiveness in resistant cells. We further examined whether MUC1 was required for TMEM59L‐induced aggressive phenotypes. Overexpressing TMEM59L increased the migration and invasion capabilities of LNCaP EnzR and 22Rv1 cells. In contrast, either shMUC1 or GO‐203 markedly attenuated these effects (Figure S4G–I). The functional importance of MUC1 was subsequently evaluated in vivo. Compared with vector tumours treated with enzalutamide, TMEM59L‐overexpressing tumours exhibited accelerated growth and increased endpoint tumour weight during enzalutamide treatment. Importantly, the addition of GO‐203 significantly suppressed the growth and final weight of TMEM59L‐overexpressing xenografts (Figure 7G–I). Collectively, these in vitro and in vivo results identify MUC1 as an important downstream effector of TMEM59L‐mediated enzalutamide resistance and malignant tumour behaviour. In addition, pharmacological inhibition of MUC1‐C with GO‐203 in NCI‐H660 cells reduced BRN2, SOX2 and ASCL1, together with the neuroendocrine markers ENO2 and SYP (Figure 7J). Similar changes were observed following MUC1 knockdown in DU145 cells (Figure 7K). The findings suggest that inhibiting MUC1 influences an extensive lineage‐regulatory program.

Research indicates that MUC1 enhances β‐catenin/TCF transcriptional activity and increases MYC expression, a pathway observed in multiple cancers.^40^, ^41^, ^42^, ^43^, ^44^, ^45^ Moreover, accumulating studies have reported that MYC is a driver of EnzR and NE differentiation in PCa.^46^, ^47^ Consequently, we investigated whether TMEM59L modulates the β‐catenin/MYC signalling network through MUC1. GSEA of TCGA‐PRAD datasets revealed that high *TMEM59L* expression is significantly associated with the enrichment of WNT/β‐catenin and MYC signalling signatures (Figure S4J). Additionally, virtual knockout of *TMEM59L* in epithelial cells, based on single‐cell data, resulted in the perturbation of 108 genes (Figure 7L). Notably, GSEA showed that these perturbed genes were characterized by downregulated WNT/β‐catenin signalling and upregulated AR response pathways (Figure 7M). Western blot analysis demonstrated that TMEM59L knockdown decreased MYC, β‐catenin, and their downstream effectors Cyclin D1 and MMP‐7, while TMEM59L overexpression increased their levels (Figure 7N). Based on these observations, we examined whether MUC1 silencing or pharmacological inhibition via GO‐203 could abrogate the TMEM59L‐induced activation of β‐catenin/MYC signalling, NE differentiation, EMT, and stemness. To this end, *TMEM59L*‐overexpressing LNCaP EnzR and 22Rv1 cells were treated with shMUC1 or GO‐203. In *TMEM59L*‐overexpressing cells, both interventions suppressed the expression of β‐catenin/MYC components, stemness markers (CD44, CD133), NE markers (ENO2, SYP) and N‐cadherin, while upregulating E‐cadherin (Figure 7O). Collectively, these findings identify the TMEM59L/MUC1/β‐catenin/MYC axis as a critical driver of enzalutamide resistance and NE differentiation, suggesting that targeting this axis represents a promising therapeutic strategy for mitigating PCa progression and overcoming drug resistance.

### 3.8 TMEM59L depletion or MUC1‐C inhibition suppresses xenograft growth and neuroendocrine‐associated signalling in vivo

**FIGURE 8:**
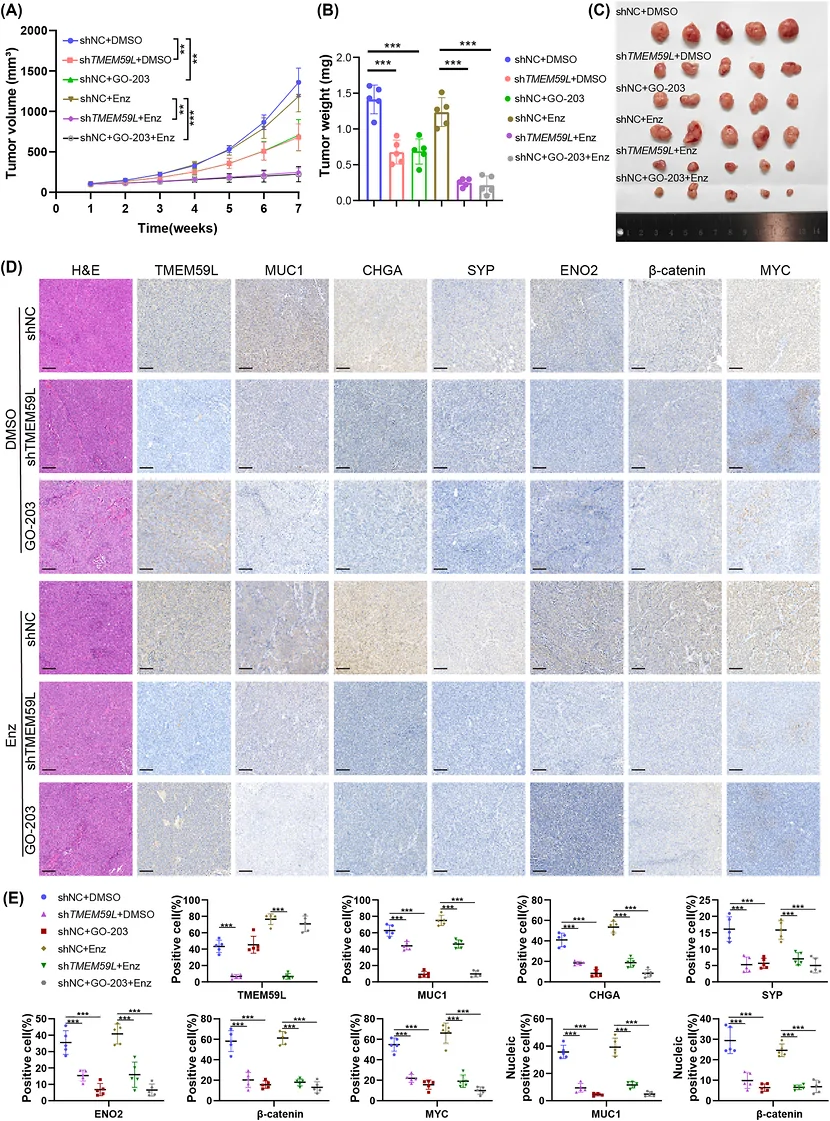
TMEM59L depletion or MUC1‐C inhibition suppresses xenograft growth, neuroendocrine‐associated features, and β‐catenin/MYC signalling in vivo. (A–C) LNCaP EnzR cells stably expressing control shRNA (shNC) or TMEM59L‐targeting shRNA (shTMEM59L) were subcutaneously implanted into male nude mice. Mice were assigned to the indicated groups and treated with vehicle (DMSO), the MUC1‐C inhibitor GO‐203, enzalutamide (Enz), or the indicated combinations (*n* = 5 per group). Tumour growth curves (A), endpoint tumour weights (B), and representative images of excised tumours (C) are shown. (D) Representative H&E staining and immunohistochemical staining for TMEM59L, MUC1, the neuroendocrine markers CHGA, SYP and ENO2, and the downstream signalling proteins β‐catenin and MYC in xenograft tumours from the indicated groups. Scale bars, 100 µm. (E) Quantification of the percentages of cells positive for TMEM59L, MUC1, CHGA, SYP, ENO2, β‐catenin and MYC, together with the percentages of cells exhibiting nuclear MUC1 or nuclear β‐catenin staining. Data are presented as mean ± SD. *p < .05; **p < .01; ***p < .001.

To validate the TMEM59L/MUC1‐C axis in vivo, we established LNCaP EnzR xenografts expressing shNC or shTMEM59L and treated the mice with vehicle, GO‐203, enzalutamide, or the indicated combinations. Both TMEM59L depletion and GO‐203 notably inhibited tumour growth and decreased final tumour weight in conditions treated with either vehicle or enzalutamide (Figure 8A–C). We next performed histopathological and immunohistochemical analyses of the harvested tumours. Immunohistochemical analysis showed that TMEM59L depletion reduced the neuroendocrine markers CHGA, SYP and ENO2, and the downstream proteins β‐catenin and MYC. GO‐203 produced similar downstream effects without substantially altering TMEM59L expression, consistent with MUC1‐C acting downstream of TMEM59L. Both interventions also decreased the proportions of cells exhibiting nuclear MUC1 or nuclear β‐catenin staining (Figure 8D,E). These findings provide in vivo evidence that inhibition of the TMEM59L/MUC1‐C axis suppresses resistant tumour growth, neuroendocrine‐associated features, and β‐catenin/MYC signalling.

## 4 DISCUSSION

**FIGURE 9:**
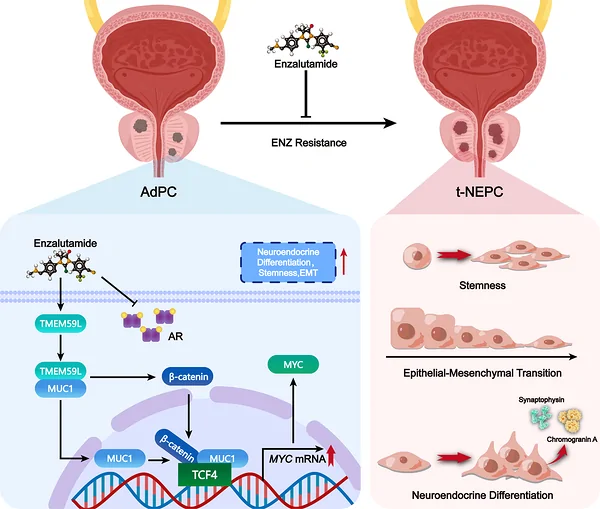
Schematic diagrams illustrating the role of TMEM59L. Enzalutamide treatment promotes the progression of adenocarcinoma prostate cancer (AdPC) towards t‐NEPC. Mechanistically, TMEM59L interacts with MUC1 and promotes its nuclear accumulation. In the nucleus, MUC1 acts as a transcriptional co‐regulator to enhance MYC transcription, thereby promoting stemness, EMT, and neuroendocrine differentiation, ultimately contributing to enzalutamide resistance.

The study highlights TMEM59L as a key factor in therapeutic resistance and neural lineage plasticity in prostate cancer. We demonstrate that *TMEM59L* is negatively regulated by AR signalling but markedly upregulated in enzalutamide‐resistant PCa. Functionally, TMEM59L promotes tumour growth, NE differentiation, EMT and cancer stemness, thereby facilitating resistance to anti‐androgen therapy. Mechanistically, TMEM59L interacts with MUC1 and promotes activation of the β‐catenin/MYC signalling cascade, establishing a TMEM59L/MUC1/β‐catenin/MYC regulatory axis that drives aggressive tumour phenotypes and therapeutic resistance (Figure 9). These findings provide new insight into AR‐independent mechanisms underlying endocrine therapy resistance in PCa and identify TMEM59L as a potential therapeutic target.

A central finding of this study is that *TMEM59L* expression is tightly linked to androgen signalling suppression and the development of enzalutamide resistance. We found that androgen stimulation significantly suppresses TMEM59L expression, whereas enzalutamide treatment markedly induces its upregulation. TMEM59L levels were notably higher in EnzR cell lines and clinical tumours exhibiting advanced clinicopathological characteristics, such as elevated Gleason scores, lymph node metastasis and reduced patient survival. Analysis of an independent tissue cohort revealed a significantly higher proportion of TMEM59L‐high specimens in the high‐Gleason‐score group compared to the low‐ or intermediate‐score groups. Multiplex immunofluorescence further localized TMEM59L predominantly to AMACR‐positive/p63‐negative malignant epithelial regions, supporting an association between TMEM59L expression and higher pathological grade in conventional prostate adenocarcinoma. These observations suggest that TMEM59L may function as a stress‐responsive molecule activated during androgen deprivation. Previous studies have shown that prolonged androgen receptor inhibition drives transcriptional reprogramming that enables tumour cells to escape AR dependency and adopt alternative survival pathways.^48^ For example, Bluemn et al. demonstrated that anti‐androgen therapy can induce lineage plasticity and promote aggressive phenotypes independent of AR signalling.^49^ Our findings extend this concept by identifying TMEM59L as a previously unrecognized component of this adaptive response, potentially serving as a molecular link between AR pathway suppression and tumour cell reprogramming.

Another important finding is that TMEM59L promotes neuroendocrine‐associated programs and lineage plasticity in PCa. Single‐cell analyses revealed a negative correlation between TMEM59L and AR activity, alongside a positive correlation with NE signatures. Consistently, TMEM59L expression correlated positively with NE scores across four advanced PCa cohorts (Beltran 2016, GSE66187, SU2C and GSE126078). Experiments demonstrated that overexpressing TMEM59L increased neuroendocrine marker expression, while its depletion reduced features associated with neuroendocrine differentiation induced by androgen deprivation. Moreover, DHT treatment failed to normalize the elevated expression of ENO2, SYP, BRN2, SOX2 and ASCL1 in TMEM59L‐overexpressing LNCaP cells. The results suggest that the neuroendocrine‐associated lineage program induced by TMEM59L is resistant to reversal by androgen stimulation. Importantly, TMEM59L knockdown in NCI‐H660 and AR‐negative DU145 cells reduced the expression of the neuroendocrine markers ENO2 and SYP and the lineage regulators BRN2, SOX2 and ASCL1, indicating that the effects of TMEM59L extend beyond individual markers to a broader lineage‐reprogramming programme. Studies by Beltran et al. and Ku et al. have highlighted the importance of transcriptional and epigenetic reprogramming in the emergence of NEPC.^35^, ^50^ Our findings therefore identify TMEM59L as a contributor to enzalutamide resistance in AR‐retaining disease and to neuroendocrine‐associated lineage plasticity in susceptible tumours.

In addition to promoting NE‐associated features, TMEM59L enhanced EMT and cancer stemness. TMEM59L overexpression increased stemness markers and mesenchymal features, accompanied by enhanced migration, invasion, and sphere formation. Given that EMT and stemness are interconnected drivers of tumour plasticity, metastasis, and therapeutic resistance,^51^, ^52^ these findings identify TMEM59L as a regulator linking these programs to anti‐androgen resistance. Importantly, these broader protumour effects indicate that increased TMEM59L expression during prostate cancer progression does not imply that all tumours will ultimately transdifferentiate into NEPC. Instead, TMEM59L may promote the progression of AR‐positive tumours through EMT, stemness, and therapeutic resistance independently of complete neuroendocrine conversion.

Mechanistically, our study identifies MUC1‐C as a functional downstream effector of TMEM59L. Co‐immunoprecipitation and GST pull‐down assays support a direct physical interaction between TMEM59L and MUC1‐C, while immunofluorescence and subcellular fractionation showed that TMEM59L overexpression increased nuclear MUC1 accumulation. Previous studies indicate that MUC1‐C nuclear import involves CQC motif‐dependent oligomerization and interactions with importin‐β and Nup62. Moreover, c‐Src‐mediated phosphorylation of the MUC1‐C YEKV motif enhances its binding to β‐catenin, while MUC1‐C can limit GSK3β‐dependent β‐catenin degradation, thereby promoting β‐catenin/TCF‐dependent transcription and activation of targets such as MYC.^53^, ^54^ Consistent with this mechanistic framework, MUC1 knockdown or GO‐203 attenuated TMEM59L‐associated β‐catenin/MYC signalling and enzalutamide resistance. In xenografts, TMEM59L depletion or GO‐203 reduced viable tumour‐cell density and produced a less compact tumour architecture, while immunohistochemistry showed attenuation of neuroendocrine‐associated features and β‐catenin/MYC signalling. Together, these findings support a TMEM59L/MUC1‐C/β‐catenin/MYC axis that contributes to lineage plasticity and drug resistance in PCa, consistent with the established involvement of MYC in therapy‐induced NE differentiation. However, whether TMEM59L directly regulates MUC1‐C processing, oligomerization, post‐translational modification, or engagement with the nuclear‐import machinery remains unresolved and will be investigated in future studies.

Nonetheless, it is important to recognize certain limitations of this study. While we present significant evidence for TMEM59L's involvement in therapy resistance, the exact upstream regulatory mechanisms governing its expression are not yet fully elucidated. While our data suggest that AR signalling negatively regulates *TMEM59L*, the transcription factors and epigenetic regulators mediating this repression require further investigation. Second, although our in vivo experiments confirm the oncogenic function of TMEM59L, additional studies using genetically engineered mouse models or patient‐derived xenografts would provide stronger evidence for its role in tumour progression. Third, although our findings highlight the importance of the TMEM59L/MUC1 signalling axis, PCa resistance is a highly complex and multifactorial process involving multiple signalling networks and tumour microenvironmental interactions. Therefore, the broader signalling landscape surrounding TMEM59L requires further exploration. Finally, because the present clinical cohort did not permit adequately powered comparisons among conventional adenocarcinoma, adenocarcinoma with NED, and pure NEPC, the relationship between TMEM59L expression and clinical lineage switching requires validation in larger, lineage‐defined cohorts.

Future studies should focus on several key directions. First, comprehensive analyses of the regulatory elements controlling *TMEM59L* transcription may reveal additional therapeutic targets for preventing therapy‐induced lineage plasticity. Second, due to the significant link between TMEM59L expression and NE differentiation, TMEM59L could be a potential biomarker for forecasting treatment resistance and disease progression. Third, our findings suggest that targeting the TMEM59L/MUC1/β‐catenin/MYC signalling axis could represent a promising therapeutic strategy. Pharmacological inhibition of MUC1 or downstream signalling components may help restore sensitivity to AR‐targeted therapies and prevent the emergence of aggressive tumour phenotypes. Ultimately, further investigation into this signalling network may contribute to the development of more effective therapeutic approaches for patients with advanced PCa.

## AUTHOR CONTRIBUTIONS

Yongjiang Yu, Xingang Cui and Xiuchen Guo conceived the project. Xiuchen Guo and Zhiwei Chen performed the in vitro and in vivo experiments. Yifan Liu and Jiacheng Huang performed bioinformatics analysis. Xiuchen Guo, Haihong Liao and Yulin Xu wrote the paper. All authors reviewed the manuscript.

## ETHICS STATEMENT

All procedures performed in this study were in accordance with the ethical standards of the Ethics Committee of XinHua Hospital and with the 1964 Helsinki declaration and its later amendments or comparable ethical standards, written informed consent was obtained from each patient.

## CONFLICT OF INTEREST STATEMENT

The authors declare no conflicts of interest.

## Supporting information



Supporting Information

## Data Availability

No datasets were generated or analysed during the current study.
