# Supplementary material for: TMEM59L promotes neuroendocrine differentiation and enzalutamide resistance in prostate cancer by regulating MUC1/β‐catenin/MYC axis
Source: Clin Transl Med. 2026 Sep 16;16(9):e70835. doi: 10.1002/ctm2.70835 (PMC13579383; doi:10.1002/ctm2.70835)
Supplement: Supplementary file 1 [file CTM2-16-e70835-s001.docx]

**TMEM59L promotes neuroendocrine differentiation and enzalutamide resistance in prostate cancer by regulating MUC1/β-catenin/MYC Axis**

**Authors：**Xiuchen Guo^1#^, Yifan Liu^1#^, Jiacheng Huang^2#^, Zhiwei Chen^1#^, Haihong Liao^1^, Yulin Xu^1^, Xingang Cui^1*^, Yongjiang Yu^1*^

^1^Department of Urology, School of Medicine, Xin-Hua Hospital Affiliated to Shanghai Jiao Tong University School of Medicine, Shanghai, 200092, People's Republic of China.

^2^Department of Urology, School of Medicine, Shanghai Children's Hospital, Shanghai Jiao Tong University, Shanghai, 200062, People's Republic of China.

^#^These authors contributed equally to this work.

^*^Correspondence to:

Yongjiang Yu Ph.D., Department of Urology, School of Medicine, Xin-Hua Hospital Affiliated to Shanghai Jiao Tong University School of Medicine, Shanghai, 200092, People's Republic of China. Electronic address: yuyongjiang@xinhuamed.com.cn

Xingang Cui Ph.D., Department of Urology, School of Medicine, Xin-Hua Hospital Affiliated to Shanghai Jiao Tong University School of Medicine, Shanghai, 200092, People's Republic of China. Electronic address: [cuixingang@xinhuamed.com.cn](mailto:cuixingang@xinhuamed.com.cn)

**Supplementary Material**

**Supplementary Figures**

**Supplementary Figure 1.**

**Supplementary Figure 1.**

(A)Survival rate curves for LNCaP EnzR and WT treated with enzalutamide. Cells were seeded into 96-well plates. After 24 hours, cells were exposed to culture medium containing different concentrations of enzalutamide. Following 7 days of treatment, cell viability was assessed using a CCK-8 assay and normalized to the drug-free condition. The calculated IC_50_ values are indicated. (B) LNCaP WT and EnzR cells were plated in 6-well plates at a density of 500 cells per well and cultured with or without 1 μM enzalutamide for 14 days. Colonies were then fixed with 4% PFA, stained with 0.1% crystal violet, and quantified using ImageJ. Clongenic survival rate were normalized to drug-free control (n = 3). Data are presented as mean ± SD. ns, not significant; ***P < 0.001. (C) Cell proliferation assessed by CCK-8 assay in LNCaP WT and LNCaP EnzR cells treated with DMSO or 1 μM enzalutamide over the indicated time points. Cell growth was measured using OD values. (D) Western blot analysis of full-length androgen receptor (AR) and the AR splice variant AR-V7 in parental (WT) and enzalutamide-resistant (EnzR) LNCaP cells. β-Tubulin served as the loading control. (E) Gene set enrichment analysis (GSEA) showing that the HALLMARK_ANDROGEN_RESPONSE gene set is negatively associated with *TMEM59L* expression. (F) tSNE visualization of single-cell RNA sequencing data from dataset GSE168668, showing clustering of cells corresponding to DMSO-treated and resistant (RESA, RESB) populations. (G) Expression analysis of *TMEM59L* across different treatment conditions, including DMSO, RESA, and RESB groups. (H) The relative gene expression of *TMEM59L* is compared between androgen-deprived LuCaP35 xenografts from castrated mice (Castration) and LuCaP35 xenografts from non-treated mice (Control) in the GSE33316 dataset. (I) Western blot analysis of TMEM59L expression in PCa tissues and paired adjacent normal tissues followed by statistical analysis. (J) *TMEM59L* transcriptional levels in TCGA_PRAD cohort with different PSA levels, different T stages and different nodal metastasis status.

**Supplementary Figure 2.**

**Supplementary Figure 2.**

(A) Gene effect scores for *TMEM59L* in prostate cancer and non-malignant cell lines. Data were extracted from the DepMap portal. Gene effect scores are derived from CRISPR knockout screens published by Broad's Achilles and Sanger's SCORE projects. Negative scores imply cell growth inhibition and/or death following gene knockout. Scores are normalized such that nonessential genes have a median score of 0 and independently identified common essentials have a median score of -1. (B) Validation of TMEM59L knockdown in LNCaP-EnzR and 22Rv1 cells transduced with a negative-control shRNA (shNC) or two independent TMEM59L-targeting shRNAs (shTMEM59L-1 and shTMEM59L-2). TMEM59L expression was assessed by qRT–PCR and Western blotting. (C) Validation of TMEM59L overexpression in LNCaP and C4-2 cells transduced with an empty vector or a TMEM59L-expressing vector. TMEM59L expression was evaluated by qRT–PCR and Western blotting. (D) Cell proliferation assessed by EdU assay in indicated cells. The EdU positive cells were qualified to analyze relative percentage in LNCaP EnzR and 22Rv1 cells in three experiments. ENZ, 20 μM enzalutamide. Data are presented as mean ± SD. *P < 0.05; **P < 0.01; ***P < 0.001.

**Supplementary Figure 3.**

**Supplementary Figure 3.**

(A) UMAP visualization of integrated single-cell RNA-seq data. Cells are colored by T stages, N stages and M stages, respectively. (B) Dot plot showing scaled expression of representative marker genes across annotated cell types. Dot size represents the proportion of cells expressing each gene, and color intensity indicates average expression level. (C) Stacked bar plots illustrating the proportions of major cell types across samples stratified by T stage, N stage, M stage, and Gleason grade group, respectively. (D) UMAP feature plot displaying the distribution of *TMEM59L* expression across all cells in the integrated single-cell dataset. (E) Expression levels of *TMEM59L* across different cell types. (F) Global cell–cell communication network inferred from ligand–receptor interaction analysis. Left: number of interactions among cell types. Right: interaction weights (interaction strength). Node size reflects interaction degree; edge thickness corresponds to communication strength. (G) Chord diagrams illustrating the DHT signaling pathway network and testosterone signaling pathway network among different cell populations, highlighting intercellular signaling relationships in the tumor microenvironment. (H) Global cell–cell communication network inferred from ligand–receptor interaction analysis using single-cell transcriptomic data from the separately extracted mCRPC sample subset. Left: number of interactions among cell types. Right: interaction weights (interaction strength). Node size reflects the interaction degree, and edge thickness represents communication strength. (I) Signaling role analysis for the Testosterone signaling pathways. Heatmaps display the relative importance of each cell type as Sender, Receiver, Mediator, or Influencer within the inferred signaling networks.

**Supplementary Figure 4.**

**Supplementary Figure 4.**

(A) Correlation analysis between TMEM59L expression and AR scores in tumor epithelial cells from single-cell RNA-seq data. The AR score was calculated using the mRNA Z score of ten AR activity genes (KLK3,TMPRSS2, PLPP1, KLK2, FKBP5, STEAP4, PART1, PMEPA1, ALDH1A3 and NKX3-1). Blue lines represent linear regression fits. (B) Correlation between TMEM59L expression and neuroendocrine marker genes (CHGA, ENO2, SYP). Upper panel: Beltran 2016 cohort. Lower panel: TCGA-PRAD cohort. All analyses demonstrate positive associations between TMEM59L and neuroendocrine marker expression. (C) TMEM59L expression levels across different spatial transcriptomic samples.

**Supplementary Figure 5.**

**Supplementary Figure 5.**

(A) Western blot analysis of MUC1 and prostate-specific antigen (PSA) in LNCaP cells treated with or without dihydrotestosterone (DHT). (B) Western blot analysis of MUC1 and PSA in LNCaP cells treated with or without enzalutamide (Enz). (C) Western blot analysis of MUC1, androgen receptor (AR), and PSA in LNCaP cells transduced with control or MUC1-targeting shRNA (shMUC1). β-Tubulin served as the loading control. (D, E) Representative colony-formation images (D) and quantification of colony numbers (E) in LNCaP EnzR cells treated with DMSO, Enz, GO-203, or Enz plus GO-203. (F) CCK-8 analysis of LNCaP EnzR cell proliferation over 96 h following treatment with DMSO, Enz, GO-203, or Enz plus GO-203. (G) Representative Transwell migration and invasion assays of LNCaP EnzR cells expressing empty vector or TMEM59L in combination with shNC or shMUC1. (H) Representative Transwell migration and invasion assays of 22Rv1 cells expressing empty vector or TMEM59L and treated with DMSO or GO-203. (I) Quantification of the migration and invasion assays shown in (G) and (H). (J) GSEA based on TCGA_PRAD cohort. Samples were stratified according to TMEM59L expression, and enrichment of MYC-related signatures and Wnt/β-catenin pathway is shown.

**Supplementary Table 1. ShRNA list**

| ShRNA | Sequence (5’-3’) |
| --- | --- |
| Sh*TM59L*-1^#^ | 5’-CCGGTGTGGCTGTTGTAGTTGTACTCTCGAGAGTACAA  CTACAACAGCCACATTTTTT-3’ |
| Sh*TM59L*-2^#^ | 5’-CCGGTATGGGCTGAGTCTGAAACACCTCGAGATACCCG  ACTCAGACTTTGTGTTTTTT-3’ |
| Sh*MUC1*^#^ | 5’-CCGGTTATACTGATTGAACTGTGTCCTCGAGAATATGAC  TAACTTGACACAGTTTTTT-3’ |

**Supplementary Table 2. qPCR primer list**

| **qPCR Primer** | **Sequence (5’-3’)** |
| --- | --- |
| *TM59L*-F | CAGCCCAGGGATTTGTCTCC |
| *TM59L*-R | CCACTATGGGCTGAGTCTGA |
| *actin*-F | CATGTACGTTGCTATCCAGGC |
| *actin*-R | CTCCTTAATGTCACGCACGAT |
| *GAPDH*-F | CTGGCCAAGGTCATCCATGAC |
| *GAPDH*-R | CTTGCCCACAGCCTTGGCAG |
